# Hierarchy of Hydrophobic and Electrostatic Interactions in DNA–Membrane Phase Selectivity

**DOI:** 10.1021/acsami.5c13271

**Published:** 2025-11-11

**Authors:** Siu Ho Wong, Yameng Lou, Yuduo Chen, Diana Morzy, Maartje M.C. Bastings

**Affiliations:** † Programmable Biomaterials Laboratory, Institute of Materials, School of Engineering, 27218 Ecole Polytechnique Fédérale Lausanne, Lausanne 1015, Switzerland; ‡ Interfaculty Bioengineering Institute, School of Engineering, Ecole Polytechnique Fédérale Lausanne, Lausanne 1015, Switzerland

**Keywords:** DNA−lipid interactions, phase-selective partitioning, hydrophobic anchoring, electrostatic bridging, multivalency, DNA nanotechnology, synthetic biology, membrane nanotechnology

## Abstract

DNA–lipid interfaces are pivotal in synthetic biology and biomedicine, yet their design for phase-separated membranes remains poorly understood. Here, we investigate how hydrophobic anchoring and electrostatic forces govern DNA partitioning in liquid-ordered (L_o_) and liquid-disordered (L_d_) lipid domains. Using programmable DNA nanostructures functionalized with hydrophobic anchors, we show that anchor hydrophobicity and chemical identity dictate binding strength and phase selectivity, while multivalency enhances affinity and preserves selective partitioning for weak anchors. Electrostatic bridging stabilizes DNA–lipid complexes but compromises specificity at high concentrations, whereas competitive monovalent ions dynamically shift equilibria toward hydrophobicity-driven localization. Dual-anchor constructs reveal hierarchical partitioning, where stronger anchors dominate despite competing preferences and the effects of multivalency. Balancing hydrophobic and electrostatic affinity is key; we establish a design hierarchy in which hydrophobic anchors control phase specificity, multivalency tunes binding strength, and ionic conditions act as secondary modulators. This work provides a roadmap for engineering responsive and phase-selective DNA–membrane interfaces, with implications for drug delivery, synthetic biology, and biomimetic DNA materials.

## Graphical abstract

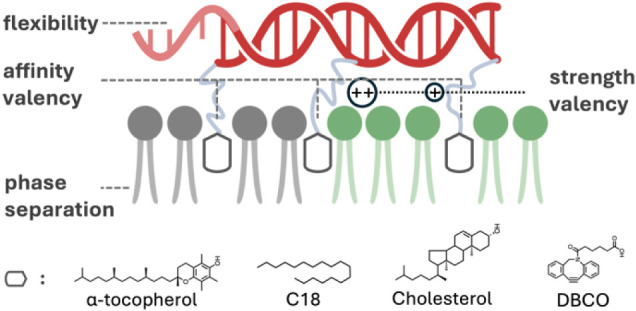

## Introduction

Nucleic acids (NA) and lipids are fundamental components of cellular systems. They also serve as critical building blocks in various synthetic nanostructures, such as vaccines,^1^ DNA nanoparticles,^2^ and artificial cells.^3^ Many of these engineered structures are designed to engage with our cells, establishing a dynamic interaction where natural and engineered systems contain the same essential molecules. This convergence generates a set of artificial interfaces vital for the consistent and predictable performance of biomaterials. Formulating a controllable NA–lipid interface demonstrates the medical benefits, facilitating advances in biomedicine. The primary examples include transforming cationic lipids for lipofection^4,5^ and exploiting lipid–DNA complexes to enhance and prolong gene expression in gene delivery.^6–8^ Particularly, the development of lipid-based NA vaccines has had positive medical and societal impacts in recent years.^9,10^

While synthetic NA–lipid systems have demonstrated significant potential, their design often relies on simplified models of lipid membranes. In reality, the NA–lipid interface is often more complex, as biological membranes are highly heterogeneous, comprising a diverse mix of lipids and proteins.^11^ This intricate asymmetry and complexity underpin essential functions, such as signaling, adhesion, and cell division.^12,13^ To organize molecular diversity, phase separation is one of the most important mechanisms, creating specialized domains or “rafts” enriched in sphingomyelins and sterols.^14,15^ The formation of distinct liquid-ordered (L_o_) and liquid-disordered (L_d_) domains selectively organizes membrane proteins, influencing processes such as signal transduction and membrane remodeling by favoring proteins of specific size and composition.^16,17^ Thus, understanding how NA interacts with these complex, phase-separated membranes is crucial for developing more sophisticated and effective synthetic systems that can mimic or interface with natural cellular environments.

In this context, the interplay of phase-separated membranes with NA molecules, such as DNA, is increasingly significant in synthetic biology. For the past decades, DNA nanostructures interacting with synthetic lipid bilayers have demonstrated great potential as a programmable tool for mimicking functionalities and biological architectures.^18^ The typical examples are to regulate transports with DNA duplexes or origami,^19,20^ to mediate cell adhesion with multivalent self-assembly of DNA,^21,22^ to sculpt membrane curvature with DNA scaffolds,^23,24^ or to construct cytoskeleton or other components of artificial cells.^25–27^ Remarkably, modified DNA nanostructures exhibit preferential partitioning into L_o_ and L_d_ coexisting synthetic bilayers, reminiscent of membrane proteins that selectively localize to lipid rafts.^28,29^ This similarity demonstrates how synthetic DNA systems replicate biologically relevant compartmentalization mechanisms found in natural membranes. The direction and degree of partitioning into different lipid phases have been reported to associate with hydrophobic anchors, membrane composition, and experiment conditions.^30,31^ However, a systematic investigation of the interplay between hydrophobic and electrostatic components^32^ – the primary drivers of DNA–lipid interactions – in phase-separated membranes remains elusive. Fundamental questions persist regarding the interplay of altering hydrophobic modifiers, the changes in their number, as well as the roles of electrostatic screening and bridging. Consequently, controlling the selective partitioning of DNA into the complex, phase-separated lipid membrane still poses a significant challenge.

In this study, we systematically investigated the mechanisms governing DNA interactions with phase-separated lipid membranes, focusing on hydrophobic anchoring and electrostatic modulation. By integrating programmable DNA nanostructures with synthetic lipid bilayers, we demonstrated that anchor hydrophobicity and chemical identity dictate phase selectivity, while multivalency amplifies binding strength for weak anchors. Electrostatic forces, mediated by divalent cations like Mg^2+^ and Ca^2+^, fine-tune binding stability and specificity, with competitive monovalent ions (e.g., Na^+^) dynamically recalibrating this balance of single- and dual-anchor systems. Our findings reveal that strong hydrophobic anchors dominate partitioning direction, even when paired with competing anchors, and that ionic conditions act as tunable “dimmer switches” to modulate DNA localization. These insights establish a design framework for engineering dynamic, phase-aware DNA–lipid interfaces, advancing applications in synthetic biology and responsive biomaterials.

## Results and Discussion

### Hydrophobic Anchoring and Electrostatic Screening Drive Phase-Specific Partitioning in Lipid Bilayers

Structurally rigid DNA can attach to zwitterionic lipid membranes in the solid/gel phase via electrostatic bridging mediated by divalent ions. They act as ionic “bridges” that link the negatively charged phosphate groups of the DNA backbone to the membrane.^33^ For liquid-phase membranes, we previously showed that DNA can be chemically modified with hydrophobic anchors, enabling robust lipid attachment independent of the lipid phase.^34,35^ Using confocal microscopy, the partitioning tendency of Cy5 fluorescent-tagged DNA (Figure S1, Table S1) to liquid-ordered (L_o_) and liquid-disordered (L_d_) phase-separated giant unilamellar vesicles (PS-GUVs) was visualized. We prepared the PS-GUVs from ternary lipid mixtures (DPPC/DOPC/cholesterol), doping at a 0.5% molar ratio of Liss Rhod-PE, which preferentially colocalizes to the L_d_ phase (Table S2). In the absence of hydrophobic modification, electrostatic “bridging” did not occur on either liquid-phase surface in 1× TE buffer (10 mM Tris, 1 mM EDTA, pH 7.5) with 0.5 mM Mg^2+^ (unless stated otherwise), even though the L_o_ phase exhibited lower membrane mobility (Figure 1a). While hydrophobic anchors (e.g., cholesterol) possessed strong affinity for lipid bilayers in the more mobile L_d_ phase, “anchoring” to both phases was prevented in the absence of divalent cations owing to inherent electrostatic repulsion between the surfaces (Figure 1b).

**Figure 1:**
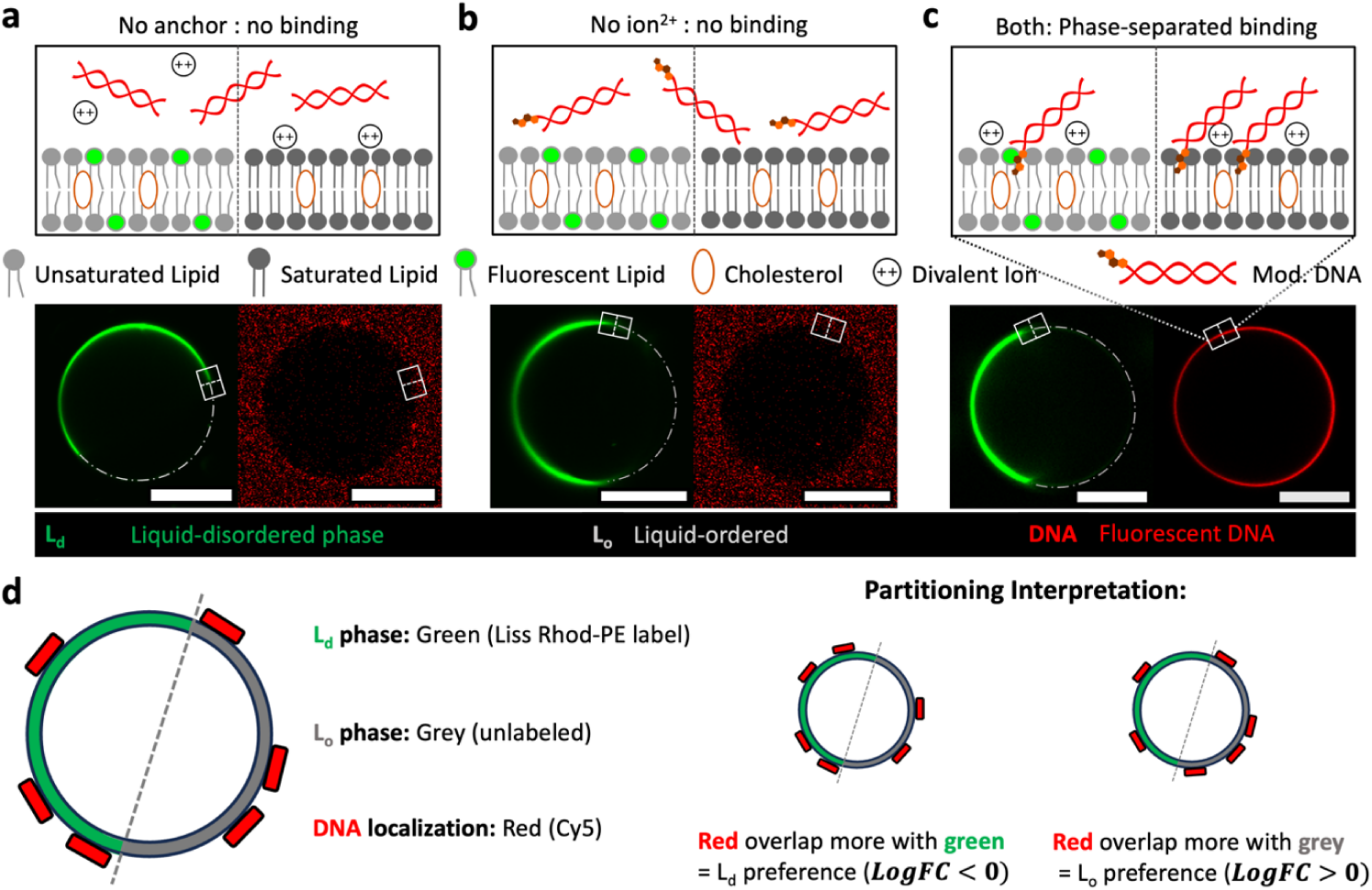
Role of hydrophobic anchoring and electrostatic screening in DNA interacting with phase-separated lipid bilayers. Experiments were conducted in 1× TE buffer (10 mM Tris, 1 mM EDTA, pH 7.5) with 0.5 mM Mg^2+^. (a) Schematic (top) illustrating that electrostatic bridging alone cannot bind or partition dsDNA (21 bp) between liquid-ordered (L_o_) and liquid-disordered (L_d_) phases without hydrophobic anchoring. Representative confocal micrographs (bottom, brightness adjusted for visualization) show DNA-functionalized phase-separated giant unilamellar GUVs (PS-GUVs) in coexistent states. The L_d_ phase (green) was labeled with Liss Rhod, and DNA duplexes (red) were labeled with Cy5. (b) Hydrophobic-mediated anchoring, here via a single cholesterol molecule, requires divalent ions for electrostatic screening to enable binding or partitioning in both phases. (c) Together, the combined effects of hydrophobic anchoring and electrostatic screening promote preferential partitioning into the L_o_ phase. (d) Legend for interpreting confocal images: The L_d_ phase is labeled green (Liss Rhod-PE), the L_o_ phase appears gray/unlabeled, and DNA is labeled red (Cy5). Partitioning is assessed by red signal overlap with green (L_d_ preference) or gray (L_o_ preference) regions and corresponding metric log FC in the ring-like GUV structures. Scale bars: 20 μm.

We hypothesized that cations could serve a regulatory role, denoted as electrostatic “screening”, not just solely essential in the L_d_ phase as we previously reported.^36^ Although divalent cations in liquid-phase bilayers do not directly bridge DNA and lipids, they regulate electrostatic interactions between molecules, thereby modulating complexation driven by hydrophobic attraction. At a low DNA-to-lipid molar ratio with Mg^2+^ screening (unless otherwise specified), DNA binding did not alter phase-separated GUV domain morphology, unlike charged lipid incorporation,^37^ indicating minimal impact on lipid phase behavior. In the presence of both anchor and divalent ion, we observed that cholesterol-modified DNA can not only attach to the membrane but also demonstrate a higher partitioning tendency into the L_o_ phase (Figure 1c). The electrostatic “screening” provided by cations enables a modified DNA’s attachment to the membrane, neutralizing their electrostatic repulsion even required to the less mobile lipids in the L_o_ phase.

For quantitative analysis, confocal images of PS-GUVs were acquired in sequential imaging between lines to avoid fluorescence crosstalk/crossover and processed using a custom-built image analysis workflow (detailed in Supporting Information, Figures S2, S3). This pipeline quantified the mean fluorescence intensities ( $I_{L_{o}}$ and $I_{L_{d}}$ ) of DNA within coexistent L_o_ and L_d_ membrane phases, respectively. The log-transformed fold change (log FC) between L_o_ and L_d_ phases, defined as $\log\mathrm{FC}=\log_{2}I_{L_{o}}/I_{L_{d}}$ (eq S1), was calculated to quantify partitioning directionality (Figure 1d). Notably, all quantitative metrics, including log FC and Selective Partitioning Index (SPI), were computed from raw, unadjusted images to ensure accurate intensity extraction, with brightness adjustments applied solely for visualization in the figures. Here, log FC serve as the quantitative metric for partitioning preference. In the presence of both hydrophobic anchors and divalent ions, the analysis yielded log FC ≈ 0.58 (>0), indicating preferential partitioning into the L_o_ phase for cholesterol-modified DNA (Figure S4a).

### Anchor Chemistry and Valency Dictate Binding Strength and Phase-Selective Partitioning

While cholesterol’s strong membrane affinity is well-established,^35,38^ its utility is limited when flexible DNA–lipid interactions are desired in a more dynamic range, such as being responsive to environmental cues. We therefore evaluated a panel of hydrophobic options that were readily accessible for DNA conjugation (Figure 2a), linked to the 5-prime of the DNA via tetraethylene glycol (TEG) spacer to facilitate the bypassing of polar headgroups and insertion into lipid membranes, as reported previously.^39^ These monovalent modifications include α-tocopherol (α-toco), also known for domain-specific membrane anchor as cholesterol (Chol),^31^ alongside underexplored molecules like octadecane (C18) and dibenzocyclooctyne (DBCO). Their hydrophobicity was categorized using partition coefficients (Log *P*) values, which indicate their distribution between hydrophobic and hydrophilic states (i.e., nonpolar and polar solvents) (Figure S4b).

**Figure 2:**
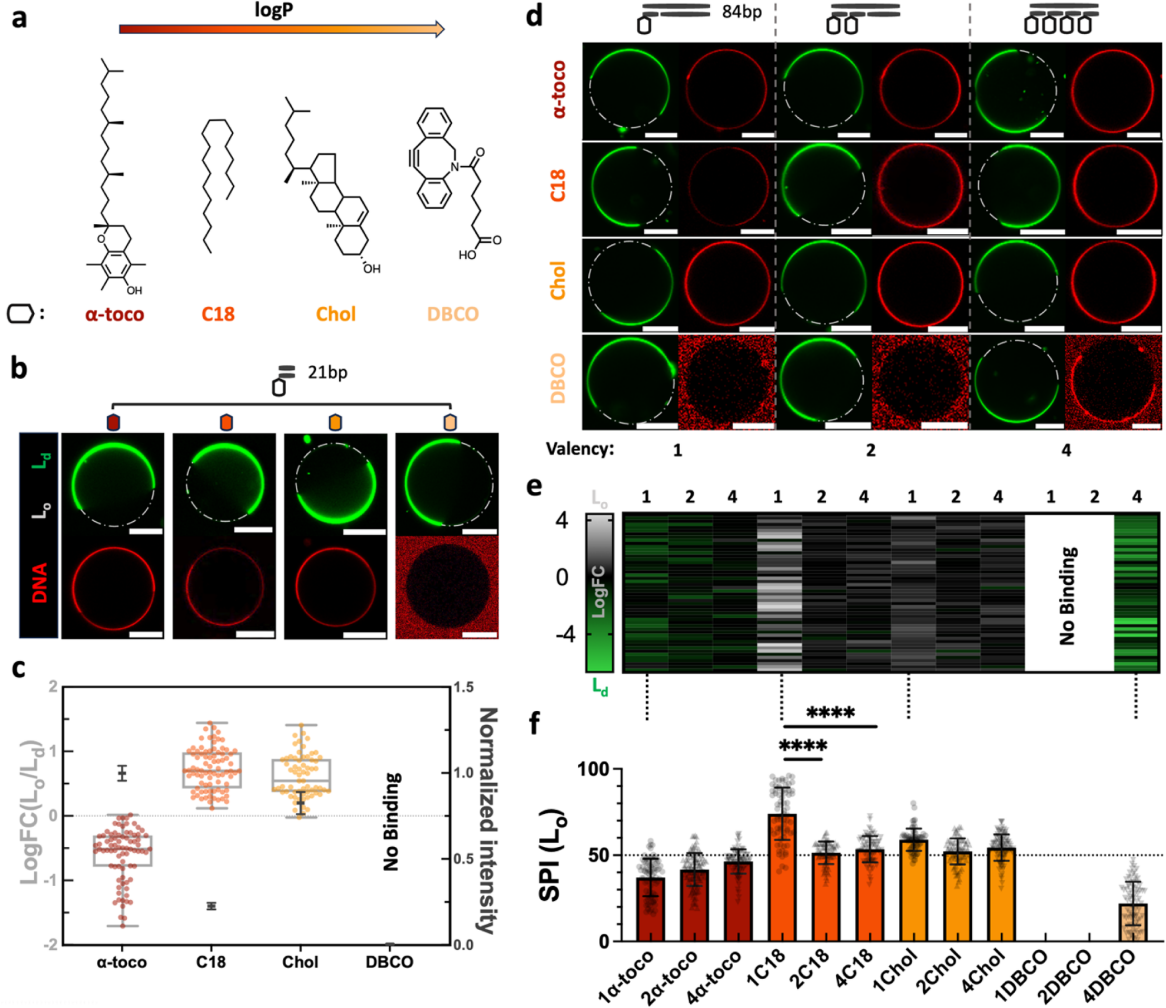
Effect of anchor hydrophobicity and valency in selective DNA–lipid interaction. (a) Chemical structures of a series of hydrophobic anchors covalently linked to DNA nanostructures. (b) Representative confocal micrographs comparing binding affinity and phase-partitioning of DNA duplexes (21 bp) with varying anchor hydrophobicity at 0.5 mM Mg^2+^. Note that image brightness for C18 and DBCO conjugates was adjusted for visualization consistency. (c) Dual-axis plots of DNA attachment to PS-GUVs, measured as the normalized fluorescence intensity (right axis) around the whole vesicles; corresponding log FC (left axis) was presented in box-and-whisker plots (*n* = 3 replicates, ≥20 GUVs per replicate). Dashed line (log FC = 0) indicating no phase preference. (d) 3 × 4 matrix of micrographs illustrating valency-dependent (left-to-right: 1 to 4 anchors) as well as hydrophobic-dependent (top-to-bottom: high to low log *P*) binding and partitioning of extended DNA conjugates (84 bp). (e) Heatmap of log FC for 50 randomly selected GUVs, with color gradients indicating L_o_ (gray) or L_d_ (green) phase tendency. (f) Bar plot of Selective Partitioning Index (SPI), combining anchor valency and hydrophobicity to evaluate the 3 × 4 matrix. Error bars represent the standard deviation (*n* = 3 replicates, ≥20 GUVs per replicate). The dashed line (SPI = 50%) denotes no preference for partitioning. Scale bar: 20 μm.

To investigate their hydrophobic binding, partitioning, and multivalency effect, we visualized these DNA conjugates (21 bp and 84 bp) with PS-GUVs at a DNA–lipid ratio (1:125 and 1:500, respectively) and 0.5 mM Mg^2+^ concentration (Figure 2b and Figure S5). It is noted that the binding efficiency and colocalization were quantified via standardized fluorescence intensity measurements. Both α-toco- and chol-modification exhibited strong membrane attachment with a directional partitioning tendency on L_d_ (log FC_α‑toco‑d21_ ≈ −0.603) and L_o_ phases (log FC_chol‑d21_ ≈ 0.612) (Figure 2c and Figure S6). Importantly, the C18 construct showed relatively moderate binding strength, dual-verified in zeta potential measurement (Figure S7), and preferential partitioning into L_o_ phases (log FC_C18‑d21_ ≈ 0.711). Similar to our previous finding, negligible interaction was observed in DBCO-modified DNA, serving as a negative control to reveal minimal background electrostatic bridging. These results reemphasize the importance of anchor hydrophobicity in enabling DNA–membrane binding, with log *P* values nonlinearly correlating to binding strength.

However, phase-selective partitioning is governed by the chemical identity of the anchor rather than hydrophobicity. α-toco and DBCO–DNA preferentially partition into the L_d_ phase due to their bulky, irregular structures that disrupt tight packing, whereas cholesterol and C18 (flexible in solution but adopting extended conformations in the L_o_ phase bilayer) favor the L_o_ phase, integrating seamlessly into densely packed, ordered lipid environments. This shape/packing argument is supported by molecular dynamics (MD) simulations and reviews of similar systems: cholesterol’s planar structure enhances L_o_ phase order by reducing lipid chain entropy and promoting tight packing,^40^ while α-tocopherol’s branched tail disrupts L_o_ domains, favoring L_d_ phases;^41^ saturated alkyl chains like C18 align with ordered lipids in L_o_ environments by minimizing gauche defects.^42^ This emphasizes the need to consider structural and functional specificity when designing lipid domain-targeted DNA interfaces.

The differential membrane-binding behavior of strong, moderate, and weak hydrophobic anchors resembles ligand–receptor affinity hierarchies.^43^ To amplify the binding of weak anchors, we utilized multivalency by clustering multiple anchors on elongated 84 bp DNA duplexes up to 4 anchors (Figure 2d and Figure S8). Binding intensities, normalized to 1-α-toco conjugate, demonstrated that 2/4-Chol, 2/4-C18, and 4-DBCO duplexes outperformed their lower-valency counterparts, confirming valency-dependent gains as anticipated (Figure S9).^32^ In contrast, strong and high-valent hydrophobic anchors (i.e., 2- to 4-α-toco/Chol) exhibited negligible multivalency effects (Figures S10, S11). Focusing on exploring the moderate and weak hydrophobic regime, including C18 and DBCO, they showed significantly enhanced binding with increased valency (Figures S12, S13). Notably, elongating the DNA duplex from 21 to 84 bp markedly improved phase-selective partitioning for the 1-C18-modified conjugate, with the tendency increased from (log FC_C18‑d21_ ≈ 0.711) to approximately 4-fold L_o_ to L_d_ phase partitioning (log FC_1C18‑d84_ ≈ 1.70) (Figure 2e). This enhancement suggests that longer duplexes, with a greater number of phosphate bridging sites, stabilize preferential phase binding when moderate anchoring alone is insufficient.

To further quantify phase-specific enrichment when accounting for membrane heterogeneity,^31^ we introduced a normalized Selective Partitioning Index: $\mathrm{SPI}=I_{L_{o}}/\left(I_{L_{o}}+I_{L_{d}}\right)\times 100\%$ (eq S2), which revealed subtle trends and focused on proportional allocation onto the L_o_ phase. For moderately hydrophobic 1-C18 conjugates, extending the DNA duplex enhanced partitioning (SPI_1C18_ ≈ 74.4%) toward the L_o_ phase via electrostatic bridging with more negative charges available. However, multimerizing C18 anchors paradoxically reduced their selectivity to 50% with no preference (Figure 2f). While strongly hydrophobic anchors also showed selectivity losses with multivalency due to overwhelming hydrophobicity, multimerizing weak DBCO anchors selectively partition (SPI_4DBCO_ ≈ 22.0%) onto the L_d_ phase. This demonstrated that anchor multimerization can compensate for low hydrophobicity while enabling tunable phase specificity. These findings establish a design framework where anchor multivalency and chemical identity jointly govern DNA–membrane interactions. While multivalency amplified binding strength, phase selectivity remained anchored to the molecular structure of the hydrophobic moiety. Integrating moderate and weak anchors with valency control offers a strategy to engineer dynamic and phase-selective interfaces beyond the limitations of conventional high-affinity anchors.

### Electrostatic Bridging and Screening Regulate Partitioning

Having established that anchor hydrophobicity and multivalency govern DNA–membrane binding and phase preference, we next interrogated how electrostatic interactions, which act in concert with hydrophobic anchoring, collectively refine these processes. Electrostatic screening by divalent cations (e.g., Mg^2+^) is a prerequisite for reducing repulsion between DNA and lipid bilayers, enabling hydrophobic anchor insertion.^34,36^ We hypothesized that subsequent electrostatic bridging and screening further stabilize DNA–lipid complexes and tune phase-specific partitioning. To dissect these contributions, we modulated (1) Mg^2+^ concentration (bridging density) and (2) cation type (bridging efficiency), focusing on moderate (1-C18) and weak (4-DBCO) anchors that exhibited the most pronounced phase selectivity in prior experiments.

Magnesium, a physiologically abundant cation central to DNA nanotechnology,^44,45^ was first tested across 0.5–2 mM concentrations, encompassing serum-relevant levels (0.75–0.95 mM).^46^ We note that the stability of the DNA constructs was not affected by changes in cation concentration (Figure S14). Increasing Mg^2+^ enhanced DNA binding for both anchors, as shown in a gradual enrichment of DNA bindings (Figure 3a, b and Figures S15–S16). Upon anchoring, elevated cation density strengthened electrostatic bridges between anionic DNA and zwitterionic lipid phosphate groups. However, this gain in binding strength inversely impacted phase selectivity for 1-C18 with SPI_1C18_ toward the L_o_ phase, dropping from ∼75% (0.5 mM Mg^2+^) to ∼56 – 53% (1–2 mM Mg^2+^) (Figure 3c, d). In contrast, 4-DBCO maintained its partitioning preference despite binding improvements, suggesting that weaker hydrophobic anchors rely more heavily on electrostatic stabilization but retain phase specificity when hydrophobicity is insufficient to dominate localization. Notably, reducing Mg^2+^ below 1 mM also destabilized strong anchors (1-α-toco, 1-Chol), diminishing their binding but increasing selectivity to SPI_1α–toco_0.2Mg_ ≈ 22.5%and *SPI*_1chol_0.2Mg_ ≈ 65.5% at 0.2 mM Mg^2+^ concentration (Figures S17–S19). This corroborates hydrophobicity as the primary driver of partitioning direction, with electrostatic forces acting as secondary modulators of binding and partitioning stability.

**Figure 3:**
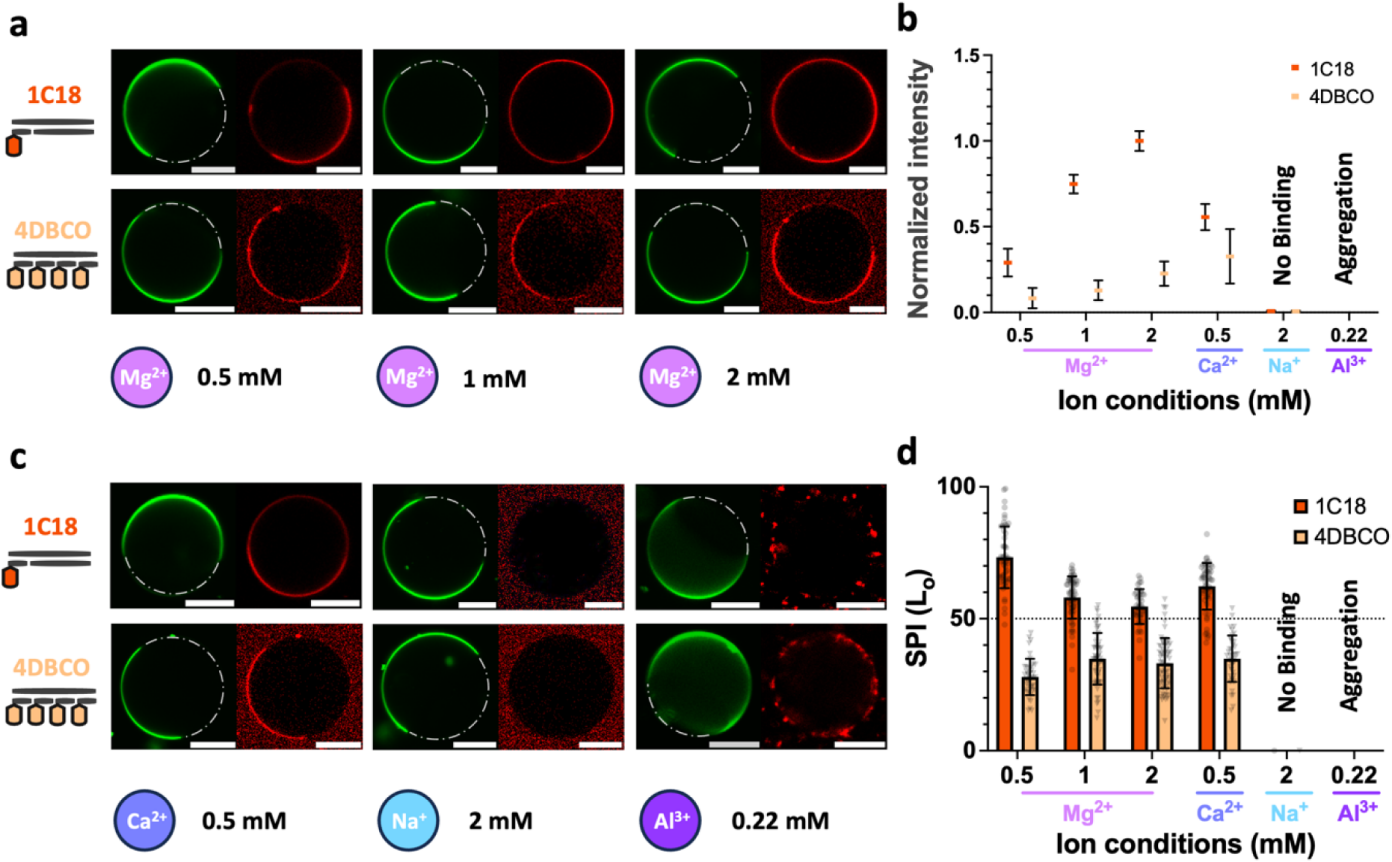
Effect of ionic strength and cation type on electrostatic bridging efficiency. (a) Confocal micrographs of 1-C18-modified DNA (84 bp) bound to PS-GUVs under varying Mg^2+^ concentrations (0.5–2 mM), qualitatively highlighting ionic strength-dependent phase selectivity. Note that image brightness was standardized, focusing on changes in partitioning. (b) Quantitative analysis of attachment efficiency for both DNA constructs to PS-GUVs under varying ionic conditions (0.5–2 mM Mg^2+^, 0.5 mM Ca^2+^, 2 mM Na^+^, 0.22 mM Al^3+^) from raw images. Binding strength correlated positively with divalent ion concentration, while Ca^2+^ exhibited stronger bridging efficacy than Mg^2+^. (c) Cation-specific binding behavior for Ca^2+^, Na^+^, and Al^3+^ at equivalent ionic strengths. Monovalent Na^+^ failed to support binding, while trivalent Al^3+^ induced DNA aggregation. (d) Selective Partitioning Index (SPI) quantifying phase preference (L_o_ vs L_d_) across ionic conditions, showing reduced specificity at higher divalent concentrations or with Ca^2+^. Error bars denote standard deviation (*n* = 2 replicates, ≥20 GUVs per replicate). The dashed line (SPI = 50%) indicates no preference. Notably, image brightness was adjusted (evident from elevated background signals) to enhance phase selectivity visualization; direct comparisons of signal intensity from qualitative images are invalid. Scale bar: 20 μm.

While Mg^2+^-mediated bridging balances binding strength and phase selectivity, the interplay of hydrophobic anchoring with other cations, varying in charge and size, remains unexplored. To study how cation identity modulates electrostatic screening and partitioning specificity, we explored the electrostatic effect of Ca^2+^, Na^+^, and Al^3+^ at ionic strengths equivalent to 0.5 mM Mg^2+^ $\left(I=\frac{1}{2}\underset{i}{\sum }c_{i}z_{i}^{2}\right)$ ,^47^ where *c*_*i*_ is the concentration of ion *i* and *z*_*i*_ is its charge, for the moderate 1-C18 conjugate. Ca^2+^, though divalent like Mg^2+^, exhibited stronger nonspecific bridging with a higher normalized signal intensity (Figure 3b, Figure S20a), as shown by the SPI_1C18_ decreasing from 75% to 62% (Figure 3d) at the same concentration. This divergence possibly arises from Ca^2+^’s lower charge density, larger ionic radius, and higher electropositivity compared to Mg^2+^, which diminish phosphate screening efficiency but serve as a stronger bridging agent to zwitterionic lipids in both phases.

Conversely, monovalent Na^+^ failed to sustain binding (Figure 3c, Figure S20b), requiring high ionic strength (50 mM) to achieve a weak attachment for moderate anchors (Figure S20c). Combined with strong anchoring (1-α-toco, 1-Chol), the Na^+^ (5 mM) screening effect was sufficient to partially preserve phase-selective binding, but higher concentrations (50 mM) induced indiscriminate adhesion (Figures S17–S19), underscoring the delicate balance between screening and hydrophobicity. Al^3+^, despite its high charge, caused DNA aggregation, likely due to excessive charge neutralization and nucleic acid condensation.^48^ Under most conditions (0.5–2 mM Mg^2+^, 0.5 mM Ca^2+^, 2–5 mM Na^+^), GUVs maintained spherical morphology and distinct L_o_/L_d_ domains, confirming stable phase separation. However, high Na^+^ (50 mM) induced tubule formation in some GUVs (Figures S18e, S19e), indicating partial membrane disruption, while Al^3+^ (0.22 mM) caused DNA aggregation without direct membrane damage (Figure S20d). These observations ensure that partitioning effects reflect anchor and ionic interactions, except at high Na^+^ or Al^3+^, where results are excluded from partitioning analyses and interpreted cautiously.

This highlights a critical trade-off: while multivalent ions enhance bridging efficiency, excessive charge density disrupts molecular integrity. These findings crystallize a hierarchy in DNA–lipid interaction design: anchor hydrophobicity and chemical identity dictate phase selectivity, while cations fine-tune binding stability and specificity. A moderate electrostatic effect (e.g., 0.5 mM Mg^2+^) optimizes partitioning precision, whereas insufficient screening compromises binding affinity and extreme bridging erodes selectivity. By decoupling anchoring from tunable electrostatic modulation, this part expands the existing toolkit for engineering programmable, phase-aware DNA–membrane interfaces.

### Competitive Interplay Modulates Binding Dynamics

Building on the role of ionic modulation, we next probed the competitive interplay between hydrophobic anchoring and electrostatic forces by engineering dual-anchor DNA nanostructures. Two constructs were designed: a “NanoPhase DNA” (NPD) with rigid spacing and a “Flexible NanoPhase DNA” (FNPD) incorporating a single-stranded DNA linker, each integrating 1-C18 (L_o_-preferring) and 4-DBCO (L_d_-preferring) anchors (Figure 4a, Figure S21). At 0.5 mM Mg^2+^ or Ca^2+^, both constructs exhibited strong L_o_-phase partitioning (SPI ≈ 70–80%), despite DBCO’s inherent L_d_ preference (Figure 4b, c and Figure S22). This aligns with 1-C18’s superior binding affinity (3-fold higher intensity; Figure 3b) and higher hydrophobicity represented by logP (Figure S4b), corroborating prior studies where stronger anchors override competing preferences,^31^ emphasizing that anchor affinity, not mere presence, governs partitioning hierarchy. The FNPD’s flexible linker slightly increased selectivity (ΔSPI ∼ 5%) while preserving binding, suggesting that a flexible hinge could inconsiderably facilitate the dominance of moderate anchors to weak anchors by increasing entropic penalties of the DNA constructs.

**Figure 4:**
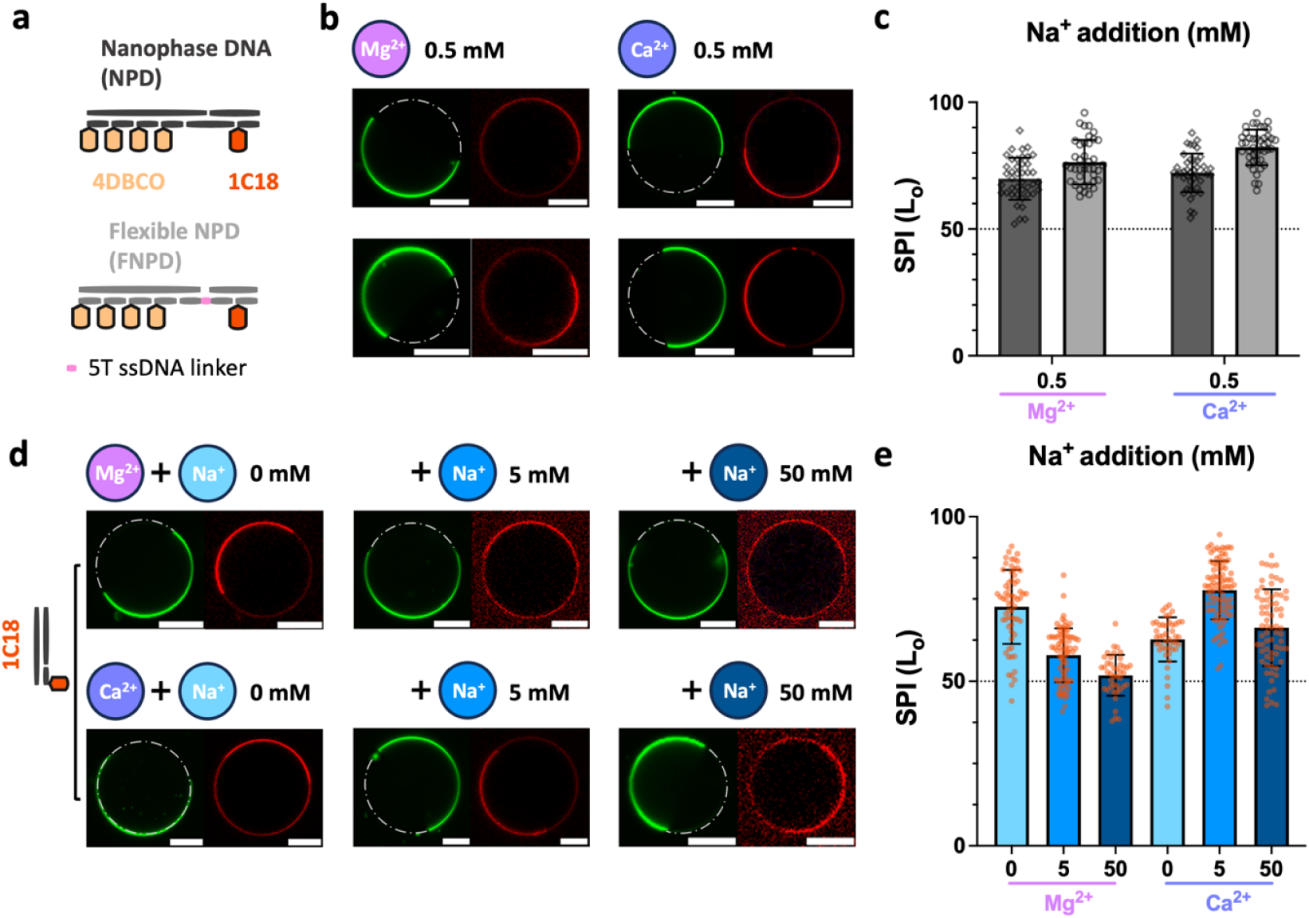
Interplay of hydrophobic and electrostatic competition in DNA–membrane interactions. (a) Schematic of dual-anchor DNA nanostructures: NanoPhase DNA (*NPD*) (rigid spacing) and Flexible NanoPhase DNA (*FNPD*) (ssDNA linker), each integrating 1-C18 (L_o_-preferring) and 4-DBCO (L_d_-preferring) anchors. (b) Representative confocal micrographs of the NPD and FNPD bound to phase-separated GUVs in 0.5 mM Mg^2+^ or Ca^2+^. (c) Selective Partitioning Index (SPI) for NPD and FNPD, quantifying phase preference. Error bars show standard deviation (*n* = 3 replicates, ≥20 GUVs per replicate). The dashed line (SPI = 50%) indicates no bias. (d) Competitive Na^+^ screening: Micrographs of the 1-C18 binding to phase-separated pre-equilibrated with 0.5 mM Mg^2+^ or Ca^2+^, followed by increasing Na^+^ addition to 50 mM. (e) Selective Partitioning Index (SPI) trends under the competitive ion conditions. The Ca^2+^ system exhibits transient selectivity gains at low Na^+^ (10:1 Na^+^:Ca^2+^), while Mg^2+^ systems decline steadily. Error bars denote standard deviation (*n* = 2 replicates, ≥20 GUVs per replicate). The dashed line (SPI = 50%) indicates no preference. Scale bar: 20 μm.

Monovalent cations, specifically Na^+^, showed weak bridging efficiency. Introducing competitive monovalent ions could be a simple route to further modulate selective DNA–membrane partitioning by reducing the number of electrostatic bridges.^49,50^ We introduced Na^+^ as a competitive ion to systems pre-equilibrated with Mg^2+^ or Ca^2+^ (Figure 4d). For Mg^2+^ -bound complexes, increasing Na^+^ addition weakened DNA attachment (Figures S23, S24a), as monovalent ions displaced divalent Mg^2+^, destabilizing electrostatic bridging. Conversely, Ca^2+^ complexes exhibited a transient increase of SPI at low Na^+^ (10:1 Na^+^:Ca^2+^ ratio) before declining at higher concentrations (Figure 4e and Figure S24b). This biphasic response likely stems from Ca^2+^’s stronger bridging efficiency. An initial low *monovalent-to-divalent ions ratio* mitigates nonspecific binding, transiently enhancing selectivity, while excess Na^+^ collapses all divalent-mediated bridges. Notably, Na^+^ competition destabilized Mg^2+^-mediated binding more readily than Ca^2+^, reflecting the latter’s higher bridging efficiency and resilience, possibly due to its lower charge density and higher electropositivity.

Further, we engineered four additional dual-anchor constructs to further probe this competitive interplay, pairing α-tocopherol (strong L_d_-preferring) with either cholesterol or C18 (strong or moderate L_o_-preferring), termed (F)­NPD_s‑s_ or (F)­NPD_s‑m_, respectively (Figure 5a, Figure S25a). Varying the *monovalent-to-divalent ion ratio* revealed distinct phase-partitioning behaviors: As we increased this ratio, similar to making it more flexible, the electrostatic bridging effect slowly weakened, leading to a lower overall binding strength (Figure S25b). For the strong–strong dual-anchor combination ((F)­NPD_s‑s_), the complex exhibited a modest L_o_ phase preference (SPI ≈ 60%) (Figure 5b, Figure S26), consistent with prior literature and likely due to cholesterol’s higher affinity to the cholesterol-rich domains.^51^ However, the introduction of competitive Na^+^ ions neutralized this preference by minimizing divalent bridging and amplifying anchor competition. This observation suggests that divalent bridging is more effective in the tightly packed lipids of the L_o_ phase compared to the loosely organized L_d_ phase. In the strong-moderate dual-anchor set ((F)­NPD_s‑m_), no phase preference emerged under high bridging conditions. As bridging weakened due to monovalent ion competition, the moderate C18 anchor’s contribution to L_o_ phase localization diminished, shifting the preference toward the L_d_ phase, driven exclusively by the α-tocopherol anchor (Figure S27). This shift persisted despite potential enhancements from multivalency and elevated local construct concentrations. Notably, monovalent ions alone failed to support binding of the moderate anchor (e.g., C18), emphasizing the critical need to balance anchor affinity and electrostatic bridging for achieving targeted selectivity.

**Figure 5:**
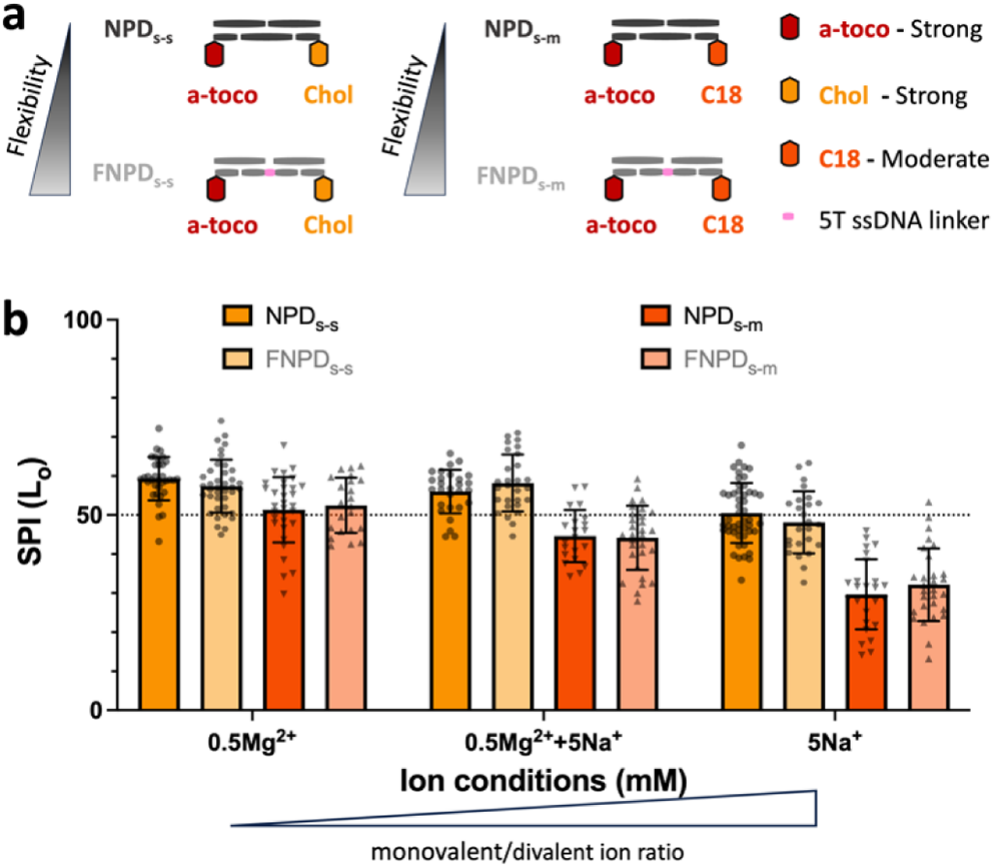
Ionic competition resets phase selectivity in dual-anchor systems. (a) Schematic of strong–strong (α-toco/chol) and strong-moderate (α-toco/C18) in the presence/absence of flexible linker NPD_s‑s_/NPD_s‑m_ and FNPD_s‑s_/FNPD_s‑m_ constructs, respectively. (b) Selective Partitioning Index (SPI) trend under varying Na^+^:Mg^2+^ ratios. Dashed line: no preference. Strong–strong anchors (α-toco/chol): High ionic bridging favors L_o_ partitioning (SPI ≈ 60%). Competition with Na^+^ (10:1 ratio) resets selectivity to neutrality (SPI = 50%; dashed line). Strong-moderate anchors (α-toco/C18): Reduced bridging shifts preference to L_d_ domains (SPI < 30%), reversing initial nonselective binding. Error bars denote standard deviation (*n* = 2 replicates, ≥20 GUVs per replicate). The dashed line (SPI = 50%) indicates no preference.

Collectively, these results suggest that competitive ions shorten the electrostatic bridge lifetime, shifting the equilibrium from stable, cation-mediated DNA–lipid tethering to a dynamic, hydrophobicity-driven binding regime. Unlike direct Mg^2+^ dilution, Na^+^ introduces kinetic competition, where frequent bridge dissociation amplifies the entropic penalty of nonspecific binding, thereby favoring hydrophobic-driven phase selectivity. This unveils an additional layer in the hierarchy of selective DNA–membrane interactions: stronger hydrophobic anchors dominate phase selectivity, while electrostatic forces act as tunable “dimmer switches” that modulate binding stability. Competitive ionic screening offers a “dynamic lever” to recalibrate this balance, given that bridging depends strongly on the monovalent-to-divalent ions ratio (e.g., Na^+^:Mg^2+^).^52^ This framework enables programmable control over DNA localization in response to environmental ion fluctuations – for example, directing DNA to the L_d_ phase under elevated Na^+^ concentrations. Such adaptability is vital for designing synthetic membranes responsive to physiological or pathological ionic cues, offering a versatile strategy for engineering responsive and phase-selective DNA–lipid interfaces.^53^

## Conclusion

This study elucidates the hierarchical principles governing DNA interactions with phase-separated lipid membranes, bridging synthetic biology and membrane biophysics. We demonstrated that hydrophobic anchors, characterized by their chemical identity and hydrophobicity (log *P*), are the primary determinants of phase selectivity. Strong anchors like cholesterol and α-tocopherol dominate partitioning direction, even in dual-anchor systems, underscoring the irreplaceable role of molecular structure in dictating membrane localization. Multivalency compensates for weak anchor hydrophobicity, enabling tunable binding strength without compromising phase specificity – a critical strategy for designing adaptable interfaces.

Electrostatic interactions, mainly mediated by divalent cations, act as secondary modulators. Mg^2+^ and Ca^2+^ stabilize DNA–lipid complexes via bridging and screening but inversely affect selectivity at higher concentrations. Higher concentrations or stronger cations (e.g., Ca^2+^) enhance binding but may reduce specificity, while monovalent Na^+^ weakens attachment unless present in excess. Competitive monovalent ions (e.g., Na^+^) introduce kinetic competition that reduces bridge lifetimes, favoring hydrophobicity-driven specificity at certain *monovalent-to-divalent ion ratios.* Crucially, ionic competition dynamically resets phase preferences – neutralizing inherent L_o_ bias in strong–strong anchor pairs (e.g., α-tocopherol/cholesterol) and inducing reversible L_d_ partitioning in strong-moderate systems (e.g., α-tocopherol/C18) by diminishing bridging effects. This extends beyond simple modulation to active reprogramming of membrane localization.

By unifying hydrophobic and electrostatic design rules, this work establishes a toolbox for engineering synthetic DNA–lipid interfaces with programmable control over membrane interactions. The demonstrated capacity to tune binding strength, phase specificity, and even reverse selectivity via ionic competition enables responsive DNA nanostructures that adapt to environmental cues. These principles advance applications from artificial cells^54,55^ to lipid raft-targeted therapeutics,^56,57^ where reversible phase-preference switching could enable context-dependent drug release. Future work should explore complex DNA architectures, partitioning kinetics, in vivo validation, as well as dedicated molecular dynamics simulations of DNA-anchored systems, to harness this hierarchy in physiological environments, where membrane complexity and environmental fluctuations demand responsive, precision-guided interfaces.

## Methods

Unless stated otherwise, chemical reagents were obtained from Sigma-Aldrich and lipids from Avanti Polar Lipids. Hydrophobic tag-TEG-DNA oligonucleotides were customized and purchased from Eurogentec with high-performance liquid chromatography (HPLC) and electrospray ionization (ESI) mass spectrometry for quality control; other oligonucleotides were from Integrated DNA Technologies, Inc. (IDT), with standard PAGE purification or HPLC purification. Chemical structures were illustrated in ChemDraw; graphs were produced using GraphPad Prism version 10.4.1 (532) for Mac.

### DNA Design and Nanostructure Assembly

All DNA nanostructures were designed and evaluated using the NUPACK suite^58,59^ to minimize secondary structure formation and optimize folding efficiency. Oligos were resuspended in Ultrapure DNase/RNase-Free distilled water to 100 μM (unmodified strands stored at 4 °C; modified strands at −20 °C). Assemblies were prepared at 1 μM final strand concentration in Tris-Eehylenediaminetetraacetic acid (EDTA) buffer (1× TE: 10 mM Tris and 1 mM EDTA, pH 8.0). Notably, strands bearing hydrophobic modifications were vigorously vortexed at 4 °C to mitigate intermolecular hydrophobic interactions. The mixtures underwent annealing in a thermocycler, starting with a 5 min incubation at 80 °C, followed by a controlled cooling from 60 to 20 °C at a rate of 1 °C per minute. The assembled structures were stored at 4 °C, and detailed information on DNA sequences and modifications is presented in Figure S1b and Table S1.

### Polyacrylamide Gel Electrophoresis

To verify the folding of designed DNA structures, native polyacrylamide gel electrophoresis (PAGE) was employed. Gels were formulated with polyacrylamide at concentrations of 10%, using 0.5× TBE buffer (Tris-Borate-EDTA, pH 8.3). The gel mixture was poured to achieve a thickness of 1 mm and left to polymerize for 30 min. Once set, the gels were transferred into an electrophoresis apparatus and submerged in 0.5× TBE buffer. DNA samples (ranging from 30 to 150 ng), along with DNA reference ladders, were loaded and run under conditions appropriate for structure length and imaged on a Bio-Rad ChemiDoc Fluorescence Imaging System.

### Phase-Separated Giant Lipid Vesicles (PS-GUVs)

Phase-Separating Giant Unilamellar Vesicles (PS-GUVs) utilized in the imaging experiments were generated using the electroformation technique, adjusted accordingly from our established protocols.^33,35,36^ The lipid mixtures consisted of DPPC (1,2-dipalmitoyl-*sn*-glycero-3-phosphocholine), DOPC (1,2-dioleoyl-*sn*-glycero-3-phosphocholine), cholesterol, and fluorescently labeled Liss Rhod PE (1,2-dioleoyl-*sn*-glycero-3-phosphoethanolamine-*N*-(lissamine rhodamine B sulfonyl)­(ammonium salt)), which preferentially colocalized into the liquid-disordered (L_d_) phase. The composition and the chemical structure of the mixtures are described in Table S2.

Lipid mixtures were deposited (80 μL, 5 mg mL^–1^) on cleaned ITO slides (15 mini sonications in EtOH, IPA, Milli-Q then N_2_ drying), desiccated 30 min, assembled with a ∼2 mm PDMS spacer and filled with degassed 200 mOsm sucrose buffer. Electroformation: The chamber was connected to a frequency generator and exposed to a 2 V sinusoidal AC (10 Hz for 2 h, then 2 Hz for 1 h). GUVs were stored at 4 °C in the dark to prevent photobleaching and used within 3 days.

### Phosphatidylcholine Assay

The concentration of synthesized GUVs was measured using a commercially available phosphatidylcholine (PC) assay kit through fluorometric detection. As per the manufacturer’s technical instructions, fluorometric signals (excitation at 535 nm and emission at 587 nm) were recorded for both the PC standard and the samples, which were diluted in a reaction mixture composed of assay buffer, hydrolysis enzyme, fluorescent peroxidase substrate, and development mix. A calibration curve was constructed using the provided PC standard solution, with the caveat that a new calibration curve must be prepared for each set of concentration measurements. To calculate the PC concentration in the synthesized vesicles, background values were subtracted from all readings, and the concentration was determined using the equation: *c* = *S*_a_/*S*_v_, where *S*_a_ denotes the amount of PC in the unknown sample (nmol) obtained from the calibration curve, and *S*_v_ represents the sample volume (μL) dispensed into the wells. Sample concentrations were adjusted to ensure consistent lipid-to-DNA molar ratios prior to the experiments.

### Confocal Microscopy

Prior to use, glass slides underwent a thorough cleaning process (15 min sonication cycles in EtOH, IPA, Milli-Q). The cleaned slides were coated with a 0.1% (w/v) solution of bovine serum albumin (BSA) to minimize nonspecific interactions and assembled into Grace Bio-Laboratories FlexWell (6.5 × 6.5 × 3.2 mm) chambers. Confocal imaging of GUVs was conducted using a Leica SP8 inverted microscope equipped with an HC PL APO 20×/0.75 dry objective. Imaging parameters were standardized to include GUVs in 1.5× (pixel sizes: 378.79 nm × 378.79 nm) to 2× (pixel sizes: 1.14 μm × 1.14 μm) zoom factor, line averaging of 4, and a controlled temperature of 25 °C (Okolab stage top chamber). Mixtures containing PC assay standardized GUVs and a DNA-to-lipid ratio of 1:125 (21 bp DNA) or 1:500 (84 bp DNA) (Figure S5, Supplementary Discussion 1) were prepared in 50 μL of a solution comprising glucose with 1× TE buffer (pH = 7.5) in a variety of ionic concentrations.

After 5 min of incubation, Liss Rhod (excitation – 560 nm; emission range – 570–630 nm) and Cy5 (excitation – 650 nm; emission range – 660–720 nm) were captured using solid-state lasers in line-by-line sequential imaging to minimize fluorescent crosstalk (Supplementary Discussion 2). Laser power and detector gain were adjusted to avoid saturation and overload. All qualitative images included in this study were processed using FIJI software,^60^ with lookup tables, display settings, scale bars, and adjusted brightness to enhance visualization. The representative micrographs were selected to qualitatively represent the average DNA binding and colocalization of specific conditions.

### Image Analysis and Additional Methods

Details of image acquisition, processing pipeline (Figure S2), vesicle detection, batch processing, and statistical analysis are provided in the Supporting Information. Preparation of large unilamellar vesicles (LUVs), together with dynamic light scattering (DLS) and ζ-potential measurements, is also described in the Supporting Information.

## Supplementary Material



## Data Availability

The image analysis pipeline code is available at https://github.com/Herbert-Wong25/Phase-separated_GUVs_Image_Analysis_2025. Large datasets, including more raw confocal microscopy images, processed images, and processed fluorescence intensity data, are available upon reasonable request due to file size constraints.

## Notes

Sequences and structures of oligonucleotides to assemble DNA duplexes; information on lipids used (details obtained from Avanti Polar Lipids); PAGE analysis and summary of DNA strands; automated image processing and analysis pipeline of DNA partitioning in GUVs; image examples for quantifying DNA nanostructure partitioning in phase-separated lipid vesicles from four randomly chosen raw images in different GUV densities; box-and-whiskers plot; saturation curves of DNA binding signal intensity; confocal images; zeta (ζ) potential measurements; quantitative analysis of attachment efficiency; polyacrylamide gel electrophoresis analysis (PDF)
