# Supplementary material for: Hierarchy of Hydrophobic and Electrostatic Interactions in DNA–Membrane Phase Selectivity
Source: ACS Appl Mater Interfaces. 2025 Nov 11;17(46):63871–81. doi: 10.1021/acsami.5c13271 (PMC12635962; doi:10.1021/acsami.5c13271)
Supplement: Supplementary file 1 [file am5c13271_si_001.pdf]

Supporting Information

Hierarchy of Hydrophobic and Electrostatic Interactions in DNA-Membrane Phase Selectivity

Siu Ho Wong[a], Yameng Lou[a], Yuduo Chen[a], Diana Morzy[a], and Maartje M.C. Bastings*[a,b]

*Correspondence: maartje.bastings@epfl.ch

Affiliations

[a]Programmable Biomaterials Laboratory, Institute of Materials, School of Engineering, Ecole Polytechnique Fédérale Lausanne, Lausanne, 1015, Switzerland

[b]Interfaculty Bioengineering Institute, School of Engineering, Ecole Polytechnique Fédérale Lausanne, Lausanne, 1015, Switzerland

Additional Methods

Image Acquisition: To evaluate DNA binding to the GUVs membranes, confocal microscopy was employed, with the fluorescence intensity of Cy5-labeled DNA serving as the key indicator. The image acquisition process followed two specific criteria to ensure data reliability. GUVs were imaged when the following conditions were satisfied:

1. The field of view contained a minimum of three phase-separated vesicles, each exceeding 20 µm in diameter. This requirement minimized the effects of Brownian motion, given that each image was captured in less than one minute.
2. The GUVs within the field of view exhibited different sizes and orientations. This ensured that they were predominantly located in a variety of focal planes and signal intensities to demonstrate a distribution of DNA binding behavior at the specific condition.
3. Minimize bright lipid aggregates, potentially resulting from imperfect electroformation, were present in or on the membrane. Observations revealed that Cy-5-labeled DNA tended to concentrate in these lipid clamps, likely due to lower entropic penalties associated with the immobile lipid environment. This accumulation necessitated careful consideration during subsequent image processing to prevent overestimation of DNA nanostructure fluorescence.

Finally, confocal images comprising two channels were acquired: Channel 1, stained with Liss Rhod-labeled lipids to visualize the liquid-disordered (L_d) phase of the GUV membranes, and Channel 2, captured Cy5-labeled DNA to access the binding of DNA nanostructures. For each experimental condition, a minimum of five images were obtained across replicates (n=3 or n=2 independent GUV preparations per condition), with ≥20 GUVs analyzed per replicate. This replicate number was chosen based on low observed variability and consistent trends in preliminary tests, as is common in GUV microscopy studies[1]. No formal p-value analysis or effect size measurements were performed; instead, claims are based on the reproducibility and directional consistency of trends (e.g., LogFC and SPI shifts) across the two replicates. Scatter plots of individual GUV data distributions are provided for visual assessment of consistency. Interpretations should be cautious due to the limited replicate number.

Image Processing: An automated Python-based image processing pipeline was developed to analyze confocal micrographs of DNA binding to the GUVs. Individual channels were separated as lipid (Channel 1) and DNA (Channel 2) channels for independent processing (**Figure S2a**). Gaussian blurring and other preprocessing operations were first applied to both channels to mitigate noise (**Figure S2b**).

- ***Vesicle Detection:*** From the OpenCV library, edge detection was first performed on the lipid channel using the Canny algorithm[2] with the customized lower and upper thresholds of 40 and 120, respectively, to delineate GUV boundaries. GUVs were identified in the preprocessed lipid channel using the Hough Circle Transform[3,4]. Parameters were optimized to detect circles with radii ranging from 10 to 100 pixels and a minimum center-to-center distance of 80 pixels, ensuring distinct vesicle identification. The function returned the coordinates and radius of detected circles (**Figure S2c**).

- ***Mask Generation:*** Subsequently, a series of masks were generated to isolate regions of interest and refine the analysis of membrane phases and DNA binding. **1)** Ring Mask: A ring-shaped region representing the GUV membrane was defined by calculating an enlarged radius (original radius multiplied by an enlargement factor of 1.2x) and an inner radius (80% of the enlarged radius). A binary mask was created to include pixels between these radii, focusing the downstream analysis on the circular membranes instead of the detected solid circles (**Figure S2d**). **2)** DNA-based Mask Refinement (Final Ring Mask): To enhance the precision of the ring mask, the DNA channel was thresholded using Otsu's method to generate a binary mask highlighting DNA-rich regions. Pixels within the initial ring mask overlapping with the DNA refining mask were extracted, and a least-squares circle-fitting algorithm was applied to these points to refine the ring mask's center and radius (**Figure S2d**). It is noted that the original ring mask was retained if fewer than three points were available.

  3) Phase-Specific Masks: Within the defined ring mask, the L_d and liquid-ordered (L_o) phases were segmented. For the L_d phase, pixel intensities in the lipid channel were thresholded using Otsu's method, and small clusters were removed via morphological operations with a minimum size of 50 pixels. The L_o phase was defined as the complement

of the Ld based on angular gap analysis, which converted the pixel positions to polar coordinates relative to the vesicle center. The dominant L_d arc is identified by the largest angular discontinuity in thresholded pixels. Notably, single-pixel L_d regions were treated as small arcs ($\pm$ 5°) to avoid computational error. Both masks were smoothed using a closing operation with a disk structuring element and skeletonized to produce single-pixel-wide representations (**Figure S2e**). These skeletons were dilated slightly (disk radius of 1) to improve statistical robustness during intensity measurements.

Intensity Measurements and Visualization: Given the final L_d and L_o phase-specific masks, the quantitative metrics were extracted for each segmented GUV to access DNA binding and phase distribution. DNA intensities from Channel 2 were measured along the dilated skeletonized masks for both phases. Importantly. Mean intensities were computed for both regions, and an overall mean intensity was calculated across the entire membrane (**Figure S2f**). To validate the pipeline's performance, four-panel visualizations were generated for each processed image. While the first panel displayed the original lipid channel with detected GUVs overloaded as red circles (**Figure S3a**), the second panel showed the labeled region ring masks (**Figure S3b**). The third and fourth panels overlaid the L_d (green) and L_o (red) masks on the lipid and DNA channels, respectively, with vesicle labels annotated for user-assisted screening as the final step of quality control (**Figure S3c, d**).

Batch Processing and Output: The pipeline was designed to process multiple images across subdirectories (conditions) within the input folder corresponding to each panel of the figures. For each image, analysis results were appended to the cumulative CSV file in the output directory, with headers included only for the first image (**Figure S2g**). This image processing pipeline provided a robust, reproducible framework for quantifying DNA partitioning to phase-separated GUV membranes, accommodating variations in vesicle size and DNA distribution. The integration of automated detection, refined masking, and detailed measurements enabled comprehensive analysis suitable for downstream data analysis and evaluation.

Data Analysis and Interpretation: First of all, the mean Cy5 (DNA) intensities of vesicles were normalized to the average intensity of a condition with the highest average intensity and

plotted as Normalized Intensity versus Different Conditions. All data was presented as means with a 95% confidence level (CI) to infer the precision of the mean.

To investigate the preferential partitioning of DNA into the liquid-ordered (L_o) and liquid-disordered (L_d) phases of GUV membranes, two complementary quantitative metrics were employed, including the Log-Transformed Fold Change ($LogFC$) and the Selective Partitioning Index toward the Liquid-Ordered Phase ($SPI(L_o)$). The $LogFC$ was determined using the formula below, representing the base-2 logarithm of the intensity ratio between the L_o and L_d phases:

$$LogFC = \log_2 \frac{I_{L_o}}{I_{L_d}} \quad \textbf{(S1)}$$

where I_{L_o} and I_{L_d} are the mean DNA intensities in liquid-ordered and liquid-disordered phases, respectively. Positive $LogFC$ values signify enrichment in the L_o phase—for instance, a $LogFC$ of 1 indicates a 2-fold higher intensity L_o compared to the L_d phase, while negative values denote a preference for the L_d phase. By symmetrizing the distribution of partitioning ratios, $LogFC$ enhances robustness against multiplicative noise, making it effective for detecting subtle directional trends across different anchors and different valencies, as shown in **Figure 2c–e**.

In addition, $SPI(L_o)$ was calculated using the formula to provide a normalized measure of relative enrichment:

$$SPI(L_o) = \frac{I_{L_o}}{(I_{L_o} + I_{L_d})} \times 100\% \quad \textbf{(S2)}$$

here I_{L_o} is the mean DNA intensity in the L_o phase, and $I_{L_o} + I_{L_d}$ is the sum of the mean DNA intensity in both phases, This metric provides a normalized value between 0 and 100, where values exceeding 50 indicate a preference for the L_o phase, and values below 50 suggest a tendency toward the L_d phase. The magnitude reflects the strength of partitioning, making its intuitive, bounded nature suitable for comparing relative DNA enrichment across experimental conditions by reflecting the proportional allocation to the L_o phase.

For analyses with ≥ 3 biological replicates, we tested normality of LogFC and SPI distributions using Shapiro-Wilk tests ($p < 0.05$ threshold) and Q-Q plots. However, statistical evaluation of

these metrics only involved hypothesis testing to determine deviations from neutrality, defined as $LogFC = 0$ or $SPI(L_o) = 50\%$. For non-normally distributed data, Mann-Whitney tests were applied. The p-values were presented as *, **, ***, **** for $p < 0.05$, 0.01, 0.001, and 0.0001, respectively. When only n=2 biological replicates were available, no inferential p-values were reported and results are described descriptively. Additionally, significant effects were identified when $LogFC$ magnitudes surpassed 1, corresponding to a 2-fold change, and $SPI(L_o)$ differences exceeded 0.2 thresholds established through prior experimental validation. To visualize partitioning direction, $LogFC$ was first plotted as box and whiskers, showing the minimum to maximum values with all data points with a dashed line at 0 to indicate no preference. Each data point represents the analysis for one measured GUVs. In **Figure 2e**, heatmaps were generated to compare directional partitioning across different multivalent anchors, using grey and green color gradients to highlight L_o and L_d preference, respectively. For $SPI(L_o)$ values were depicted in a scatter plot with error bars to demonstrate the mean value with SD along with a dashed line at 50 to indicate no preference.

The dual-metric approach was adopted to leverage the strengths of both measures. $LogFC$ provides a statistically rigorous assessment of directional partitioning, resolving subtle differences across experimental conditions, as illustrated in **Figure 2c, 2e**. $SPI(L_o)$ offers an accessible, bounded measure of partitioning magnitude, ideal for assessing relative enrichment in scenarios with pronounced or minimal partitioning preferences, as seen in **Figure 2e** onwards. Together, $LogFC$'s sensitivity to fold-change magnitudes and $SPI(L_o)$'s focus on proportional allocation ensure a comprehensive analysis, mitigating the impact of outliers and skewed intensity distributions. To control for DNA binding variability, all DNA intensity measurements were normalized to the global mean DNA intensity of the strongest binding condition.

Large Unilamellar Vesicles (LUVs): Large unilamellar vesicles (LUVs) used for ζ-potential measurements were prepared via extrusion using a commercial extruder (Avanti® Polar Lipids protocol). Size uniformity was achieved using 100 nm Whatman® Nucleopore Track-Etched Membranes and Filter Supports. A lipid film of DMPC (1,2-dimyristoyl-sn-glycero-3-phosphocholine) was formed in a round-bottom flask and rehydrated with a solution of 200

mM sucrose in TE buffer (pH 7.5). Extrusion was conducted at 40 °C to ensure bilayer fluidity. Dynamic light scattering (DLS) confirmed the LUV size distribution. Prepared LUVs were stored at 4 °C and utilized within one week.

Dynamic Light Scattering (DLS) and ζ-Potential Measurements: LUV hydrodynamic radii and ζ potentials were measured using a Zetasizer Nano ZSP (Malvern Panalytical) with a 633 nm excitation wavelength and a fixed 173° scattering angle. For size analysis, samples were loaded into a DTS1070 cell cuvette (Malvern Panalytical), and intensity-based hydrodynamic profiles were recorded as quality control. ζ-potential measurements were performed in 1 mL of 200 mM sucrose containing 1 mM Mg^{2+} in 1x TE buffer (pH 7.5) at 1:125 DNA-lipid molar ratios (see saturation curves in **Figure S5**). Absolute ζ-potential changes ($|\Delta\zeta| = |\zeta_t - \zeta_0|$) were calculated by subtracting the baseline ζ-potential of naked DMPC LUVs (in 1 mM Mg^{2+}). All other measurements were conducted at 37 °C after 5 min of thermal equilibration. Data represent averages of ≥3 independent measurements per condition, with each measurement comprising ≥15 sub-runs.

Supplementary Discussion 1

Effect of highly charged DNA on lipid phases: While the highly charged nature of DNA could potentially influence lipid phase separation through electrostatic interactions, as demonstrated in systems with incorporated charged lipids, we observed no such effects in our experiments. Visual inspection of PS-GUVs before and after DNA binding revealed consistent phase domain morphology, with no changes in domain size, shape, or stability. This lack of perturbation is attributable to the external surface binding of DNA via hydrophobic anchors, rather than incorporation into the bilayer, combined with our DNA-to-lipid molar ratio and the presence of Mg^{2+} for electrostatic screening, which minimizes any potential disruption to lipid packing or phase behavior. Similar observations have been reported in other DNA-lipid conjugate studies at low concentrations, supporting the validity of our partitioning analyses.

Supplementary Discussion 2

Time delay in sequential imaging: We acknowledge that sequential imaging could introduce a possible time delay between the acquisition of images for different fluorophores because the microscope must switch excitation wavelengths and emission filters for each channel. For the adopted line-by-line sequential imaging, each line of the image was scanned for all fluorophores before moving to the next line. This reduced the time delay significantly compared to frame-by-frame imaging. According to Pawley's handbook of confocal microscopy[5], confocal systems often use fast components like acousto-optic tunable filters (AOTFs), which can switch laser lines in about 10 to 50 microseconds, while slower mechanical parts, such as filter wheels, may take 1 to 5 milliseconds. Since the Leica TCS SP8 employs an AOTF for laser switching and can adjust settings like exposure time, its channel-switching delay aligns with the handbook's range. The delay between capturing different fluorophores for the same line is on the order of **microseconds to milliseconds**, depending on the speed of the laser scanning and filter switching and image settings (e.g., exposure time, resolution). Therefore, we assumed that misalignment or misrepresentation of the spatial and temporal relationships between the labeled DNA and GUVs is negligible.

Supplementary Figures

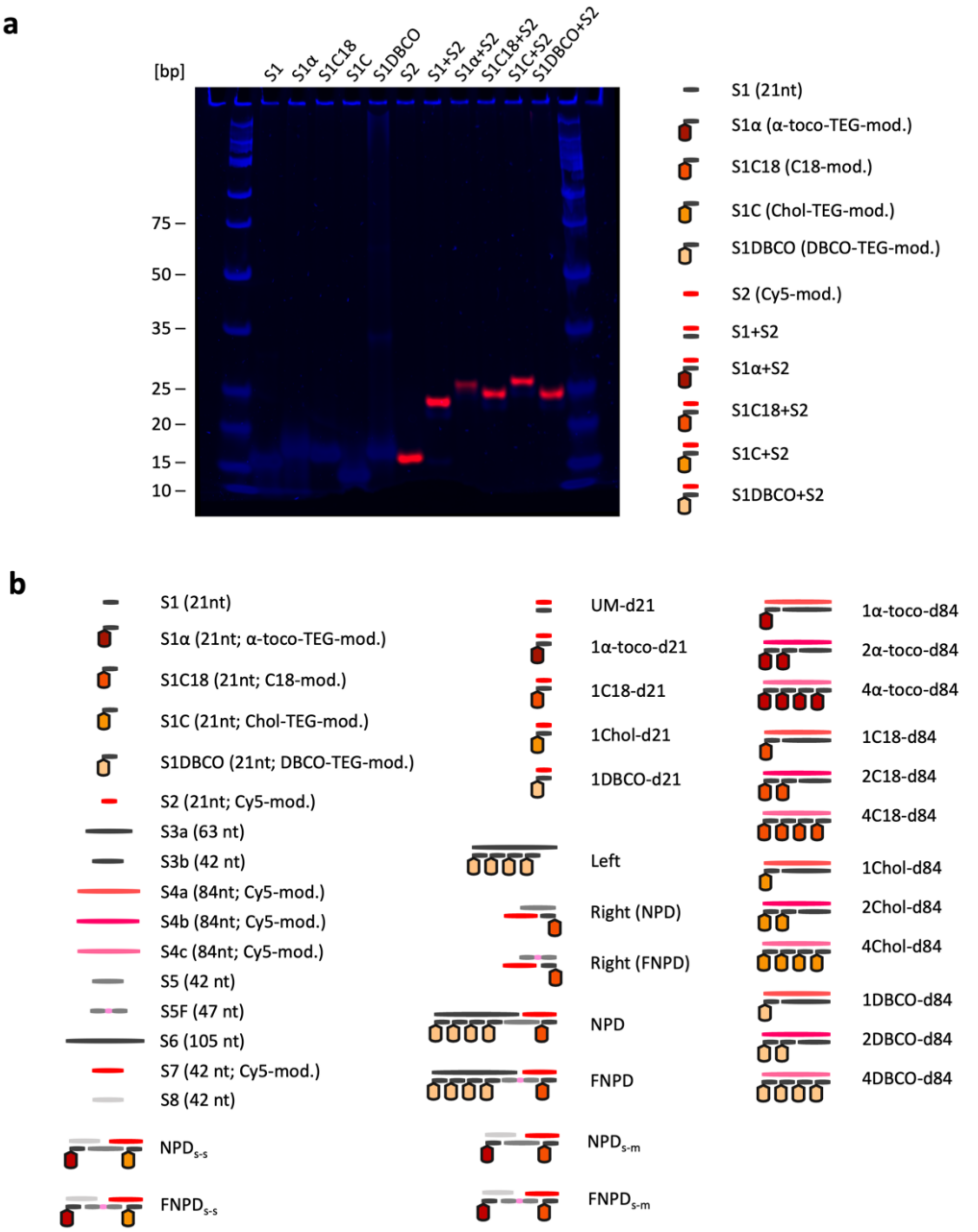

Figure S1. (a) Polyacrylamide gel electrophoresis (PAGE) analysis and summary of DNA strands. PAGE (*left*) showing unmodified and anchor-modified 21 bp-long DNA duplex labeled with Cy5 at 0.5 mM Mg^{2+}. Schematic drawings (*right*) illustrating the corresponding strands described in Table S1 and the assembled structures. (b) Schematic illustration of all strands and their visual representations.

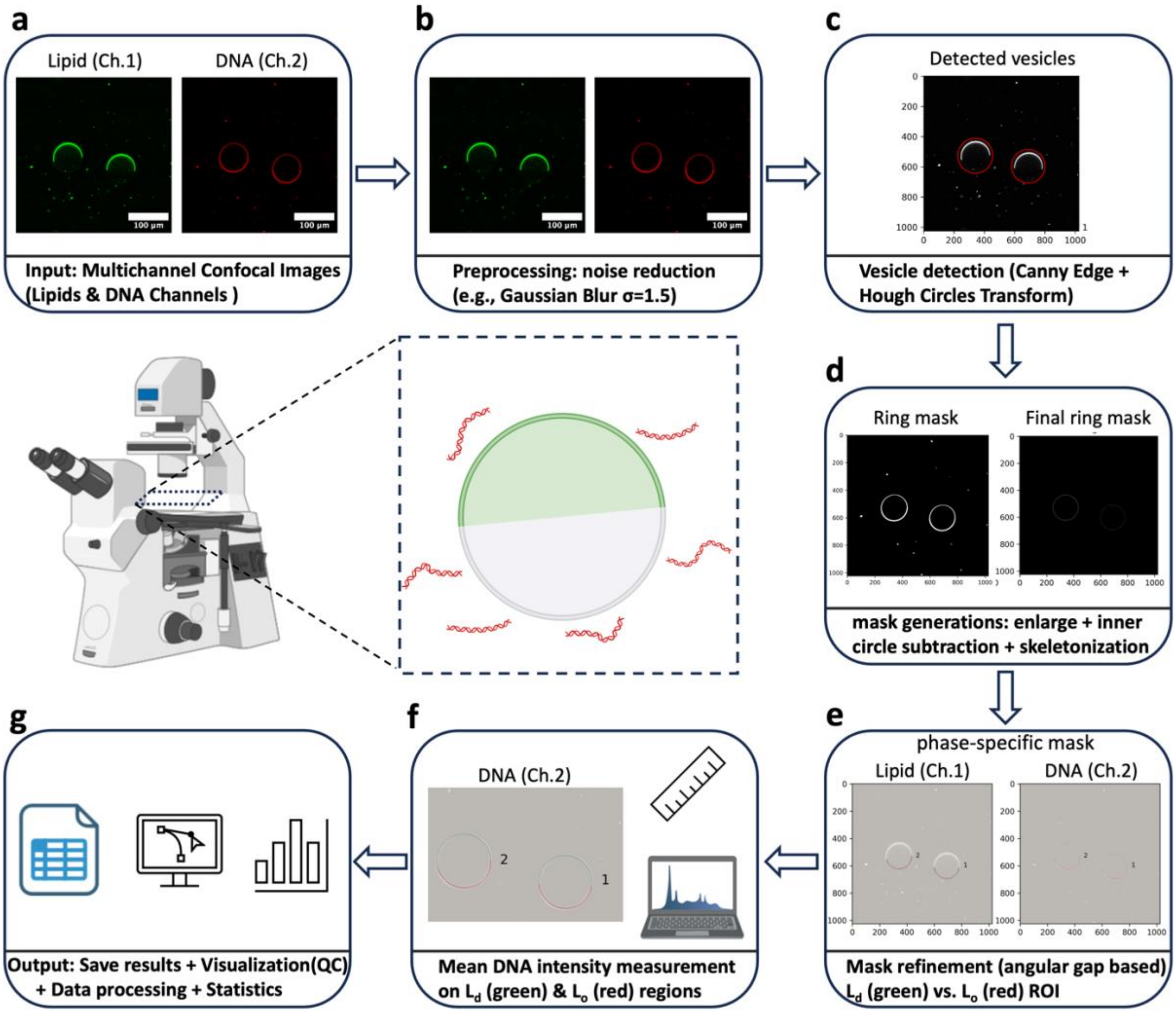

Figure S2. Automated image processing and analysis pipeline of DNA partitioning in GUVs. The flowchart illustrates the high-level stages of the automated image analysis pipeline used to quantify the partitioning of Cy5-labeled DNA between liquid-ordered (L_o) and liquid-disordered (L_d) membrane phases in GUVs. (a) Starting with multichannel confocal images (lipid channel: Liss Rhod in green; DNA channel: Cy5 in red), (b) the pipeline involves preprocessing to enhance image quality, (c) detection of GUVs using the Hough Circle Transform, (d–e) generation and refinement of masks to segment L_o and L_d phases, (f) measurement of DNA fluorescence intensities and phase areas, (g) visualization of results in multi-panel plots, and output of quantitative data and figures. Each stage includes placeholders for representative images, such as confocal micrographs, detected vesicle outlines, segmented phase masks, or final visualization plots, which can be customized to reflect experimental outputs. Note that (c–e) axes present pixel dimension in 1.5x zoom factor (pixel sizes: 378.79 nm × 378.79 nm) rather than image sizes in (a,b). Created in BioRender. https://BioRender.com/ gxf45vs.

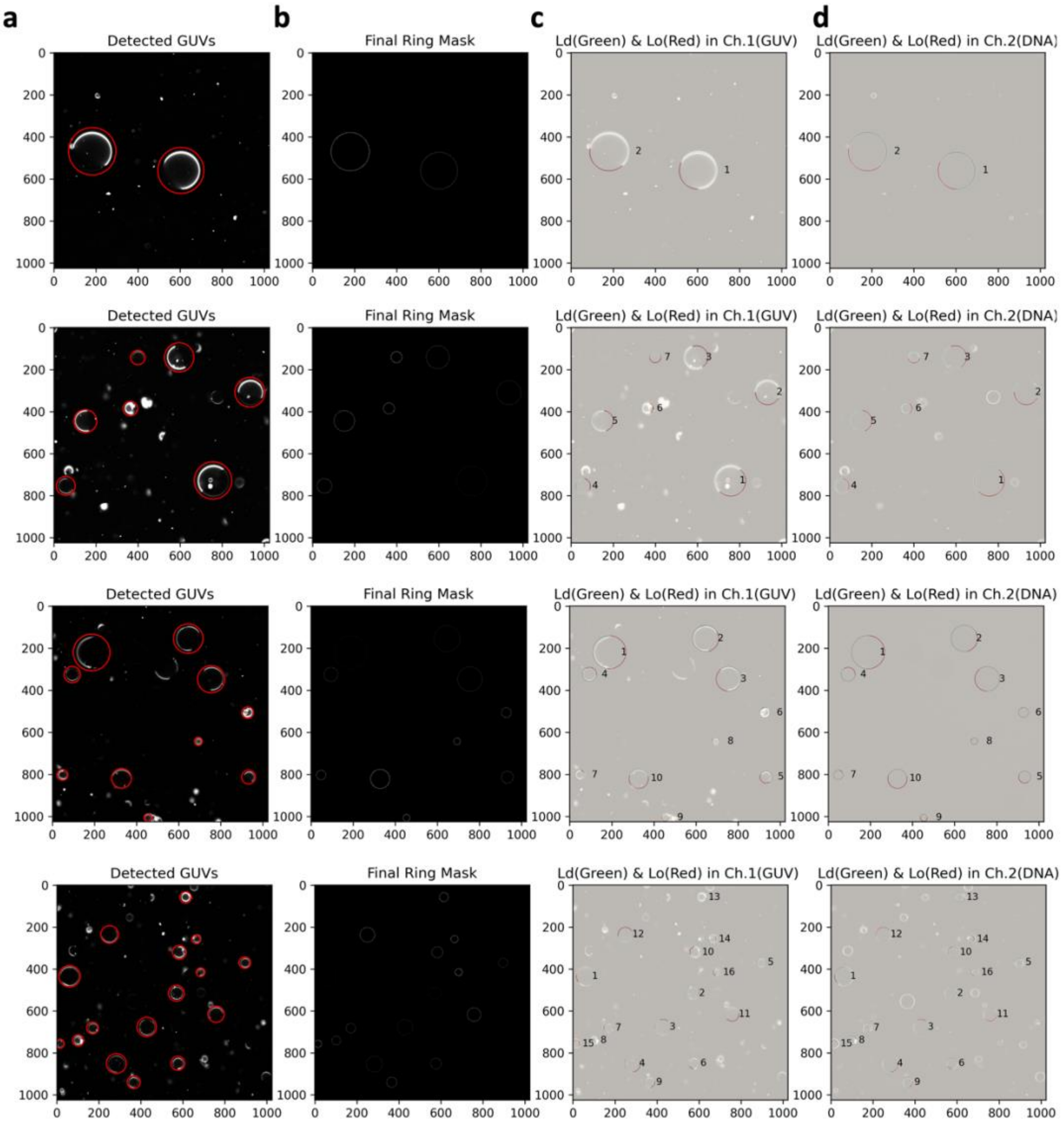

Figure S3. Image examples for quantifying DNA nanostructure partitioning in phase-separated lipid vesicles from four randomly chosen raw images in different GUV densities. (a) Vesicle detection and region of interest (ROI) definition. Confocal micrographs of lipid membranes (Liss Rhod-PE, Ch. 1) and DNA nanostructures (Cy5, Ch. 2) are acquired sequentially (inter-channel delay <1 ms) to minimize cross-talk. Lipid channel images are preprocessed with Gaussian blurring and Canny edge detection (thresholds: 40–120) to enhance vesicle boundaries. Hough Circle Transform was applied to detect vesicles (radius range: 10–100 pixels), generating circular ROIs (red outlines). (b) Final ring mask generation from DNA intensity-based refinement mask by thresholding Ch.2 (Cy5) using Otsu's method, followed by size filtering. Final ring masks (while) are derived from the intersection of expanded vesicles ROIs and DNA masks, followed by skeletonization and least-squares circle fitting to refine geometric parameters. (c) Overlay of L_d-phase mask (green) and L_o-phase mask (red) and lipid channel (Ch.1) fluorescence, verifying spatial alignment of the start and end points of segmented phase-separated regions with lipid membrane features for quality control. (d) Overlay of coexistent-phase mask and DNA channel (Ch. 2) fluorescence highlights DNA enrichment in both regions. Note that user-assisted screening excluded vesicles with incomplete segmentation or ambiguous phase boundaries prior to intensity quantification. Parameters are detailed in Methods—*Mask Generation*.

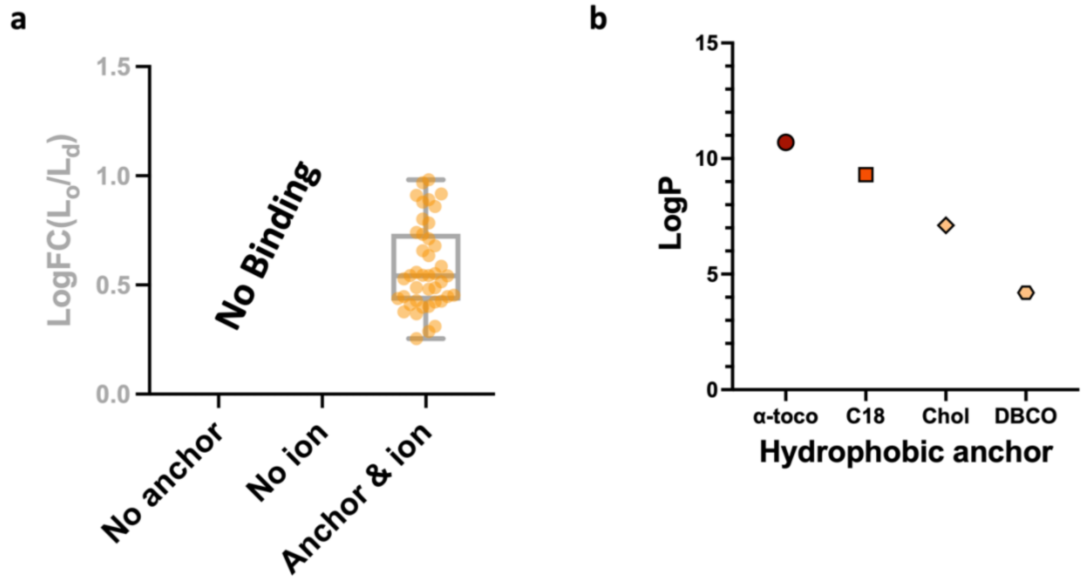

Figure S4. (a) Box-and-whiskers plot of the Log-Transformed Fold Change ($LogFC$) in DNA partitioning between L_o and L_d (n = 2 replicates, ≥ 10 GUVs per replicate). A $LogFC$ of 0 indicates no partitioning preference. (b) Categorization of hydrophobic anchors, including α-tocopherol (α-toco), octadecane (C18), cholesterol (Chol), and dibenzocyclooctyne (DBCO) in this study based on their partition coefficients (LogP), calculated using Chemicalize®[6]. LogP quantifies a compound's distribution between hydrophobic (non-polar) and hydrophilic (polar) phases, as exemplified by its partitioning in non-polar and polar solvents.

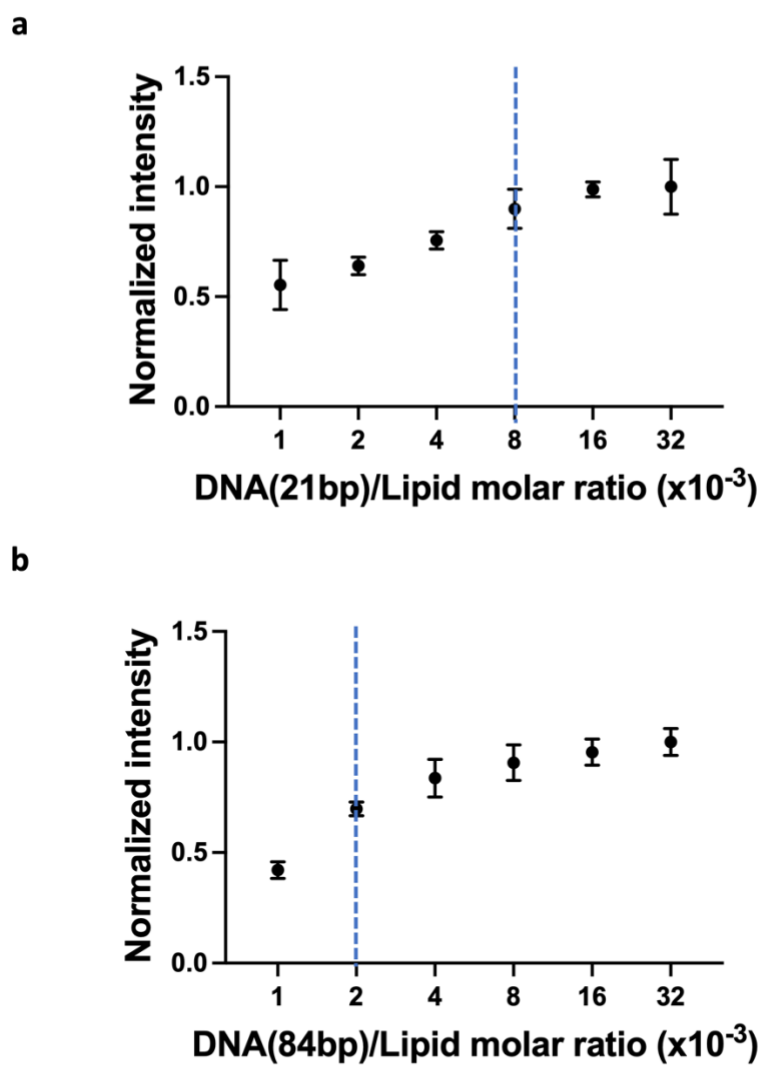

Figure S5. Saturation curves of DNA binding signal intensity conveyed by plots of means with 95% CI for phase-separated giant unilamellar vesicles (PS-GUVs) decorated with (a) cholesterol-modified DNA (21 bp) and (b) 4-cholesterol-modified DNA (84 bp) at increasing nominal DNA/lipid molar ratios, and thus resulting in different binding densities of DNA constructs to bilayers. The molarity of GUVs was determined using the phosphatidylcholine (PC) enzymatic assay described above. Dashed lines represent the adopted DNA/lipid molar ratios of 1:125 and 1:500 to avoid membrane saturation of 21 bp and 84 bp DNA, approximately 1.80×10^8 and 4.5×10^7 84 bp DNA molecules per vesicle, respectively.

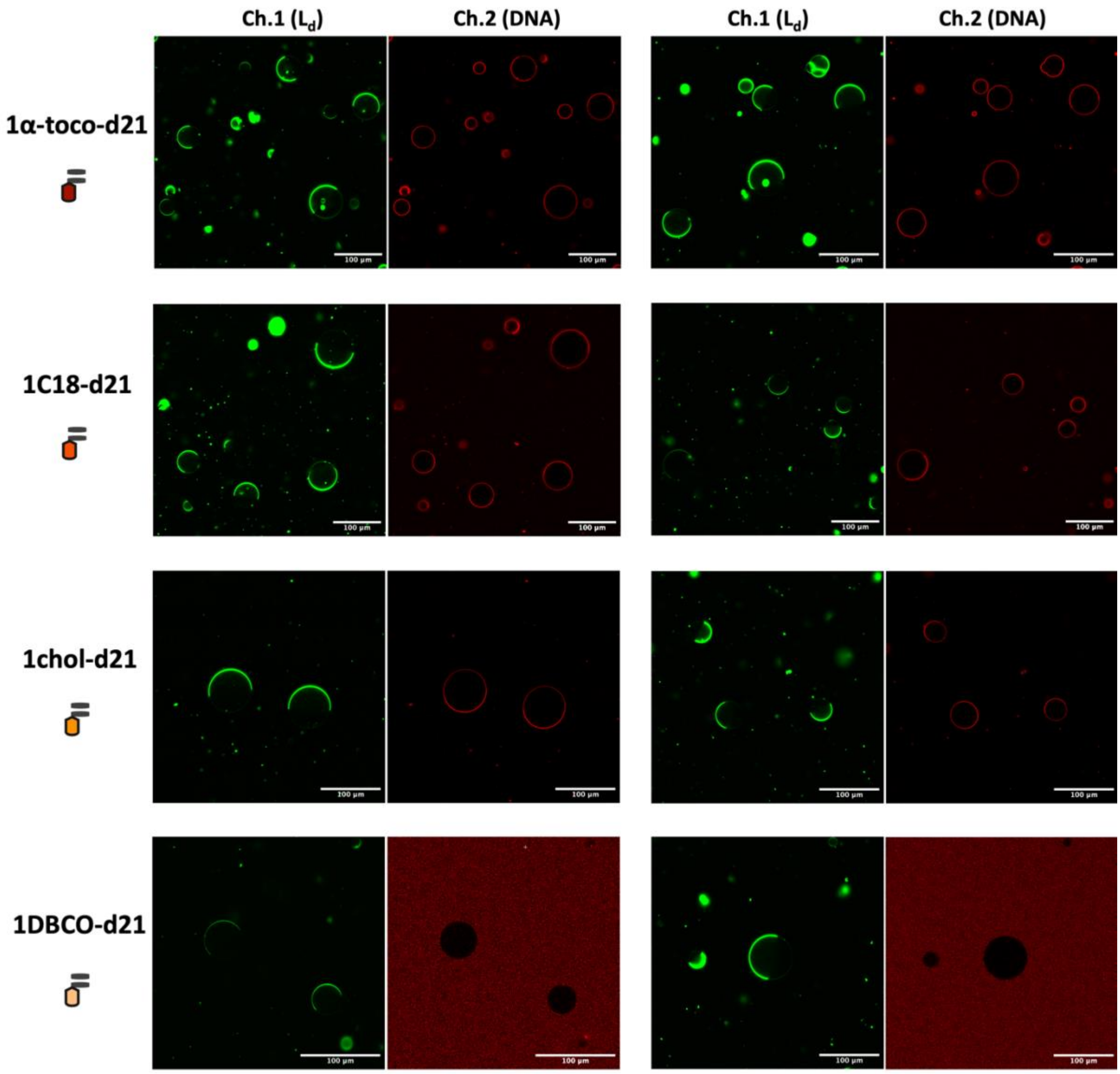

Figure S6. Confocal microscopy images of modified DNA (21 bp) with hydrophobic anchors in 0.5 mM Mg^{2+} qualitatively illustrate their distributions of binding and phase partitioning to PS-GUVs (i.e., preferential localization to L_o or L_d phase). Shown (top to bottom): single α-tocopherol (α-tocol), single octadecane (C18), single cholesterol (Chol), and single dibenzocyclooctyne (DBCO). Brightness for the images of C18 and DBCO constructs was increased by 70% and 90%, respectively, to improve the visualization of phase selectivity (evident from elevated background signals). Direct signal intensity comparisons between groups are invalid. Imaging parameters (laser intensity and gain) were standardized using the 1-α-tocopherol sample as a reference and optimized to achieve minimal saturation around GUVs. These randomly selected images supplement the representative micrographs in **Figure 2b**. Scale bar = 100 μm.

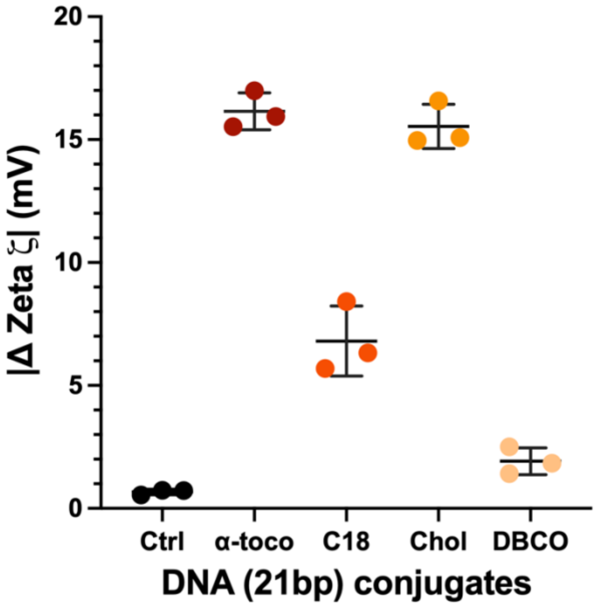

Figure S7. Zeta (ζ) potential measurements of single-hydrophobically anchored DNA (21 bp) binding to liquid-phase DMPC large unilamellar vesicles (LUVs), demonstrating the moderate binding affinity of the C18 anchor compared to strong anchors. Error bars indicate the standard deviation from three replicates, each consisting of three independent measurements (≥15 sub-runs per measurement).

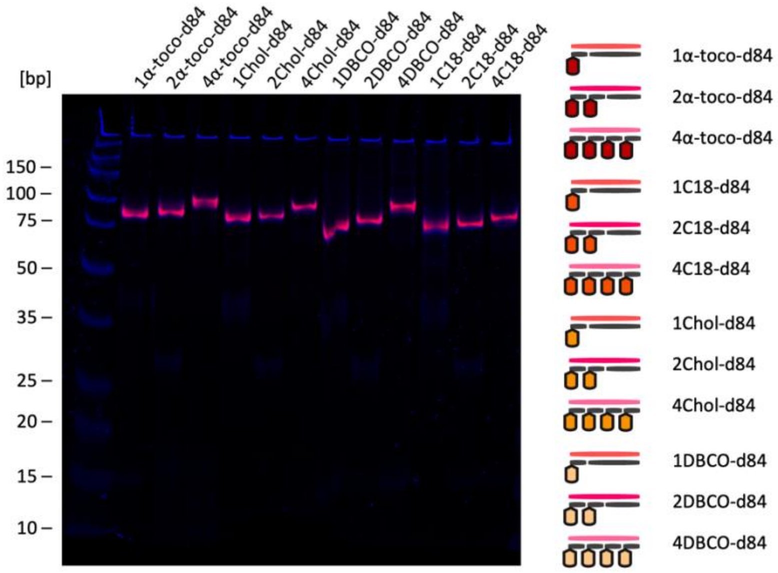

Figure S8. Polyacrylamide gel electrophoresis analysis of anchor-modified 84 bp long DNA duplex labelled with Cy5 at 0.5 mM Mg^{2+} and schematic drawings illustrating the corresponding strands described in Table S1 of the assembled structures.

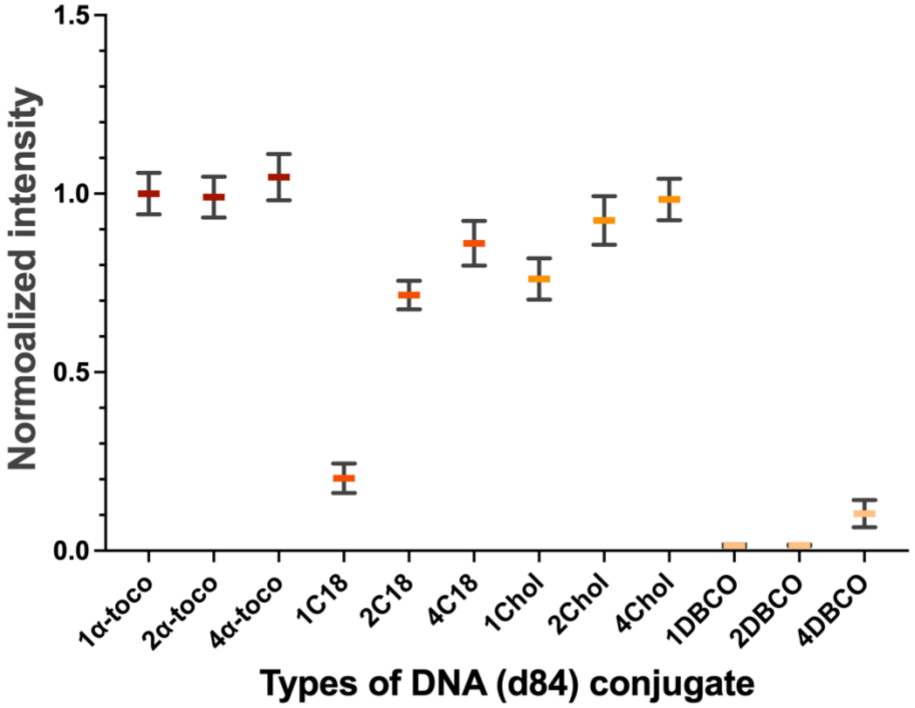

Figure S9. Quantitative analysis of attachment efficiency for multivalently modified DNA (84 bp, 1–4 hydrophobic groups) to phase-separated giant unilamellar vesicles (PS-GUVs) in 0.5 mM Mg^{2+}. Hydrophobic modifications include α-tocopherol (α-toco), octadecane (C18), cholesterol (Chol), and dibenzocyclooctyne (DBCO). Fluorescence signal intensities of all DNA nanostructures were normalized to the single α-tocopherol-modified DNA (reference value = 1). Data points represent mean values with 95% confidence intervals (CI), derived from two replicates (≥20 vesicles per replicate).

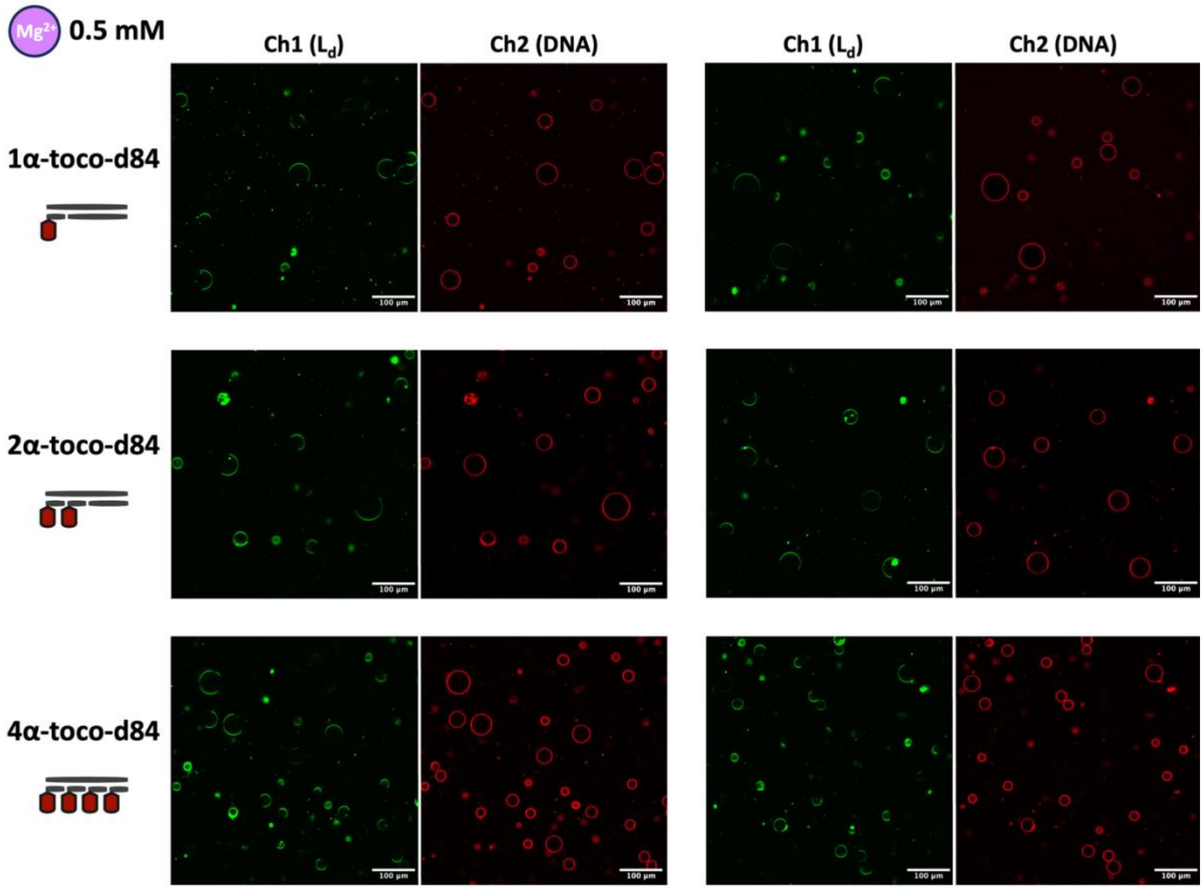

Figure S10. Confocal images of multivalently modified DNA (84 bp, 1–4 α-tocopherol) binding to PS-GUVs, qualitatively demonstrating binding and partitioning distributions in 0.5 mM Mg^{2+} (unless otherwise specified). Shown (top to bottom): 1-α-tocopherol (1α-toco), 2-α-tocopherol (2α-toco), and 4-α-tocopherol (4α-toco) modifications. Imaging parameters (laser intensity and gain) were standardized using the 4-α-tocopherol sample as a reference and optimized to achieve minimal saturation around GUVs. These randomly selected images supplement the representative micrographs in **Figure 2d (*α-toco group*)**. Scale bar = 100 µm.

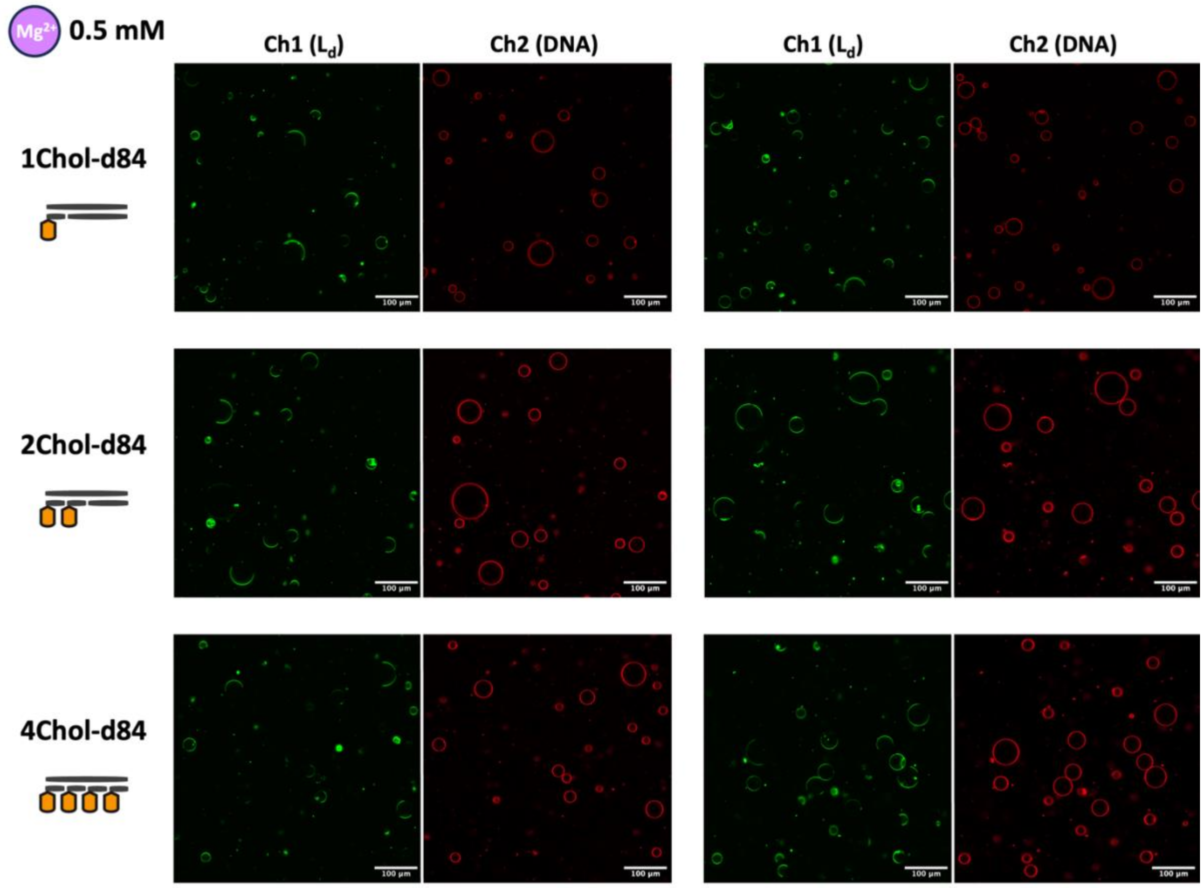

Figure S11. Confocal images of multivalently modified DNA (84 bp, 1–4 cholesterol) binding to PS-GUVs, qualitatively demonstrating binding and partitioning distributions. Shown (top to bottom): 1-cholesterol, 2-cholesterol, and 4-cholesterol modifications. Imaging parameters (laser intensity and gain) were standardized using the 4-α-tocopherol sample as a reference and optimized to achieve minimal saturation around GUVs. These randomly selected images supplement the representative micrographs in **Figure 2d** (***Chol group***). Scale bar = 100 μm.

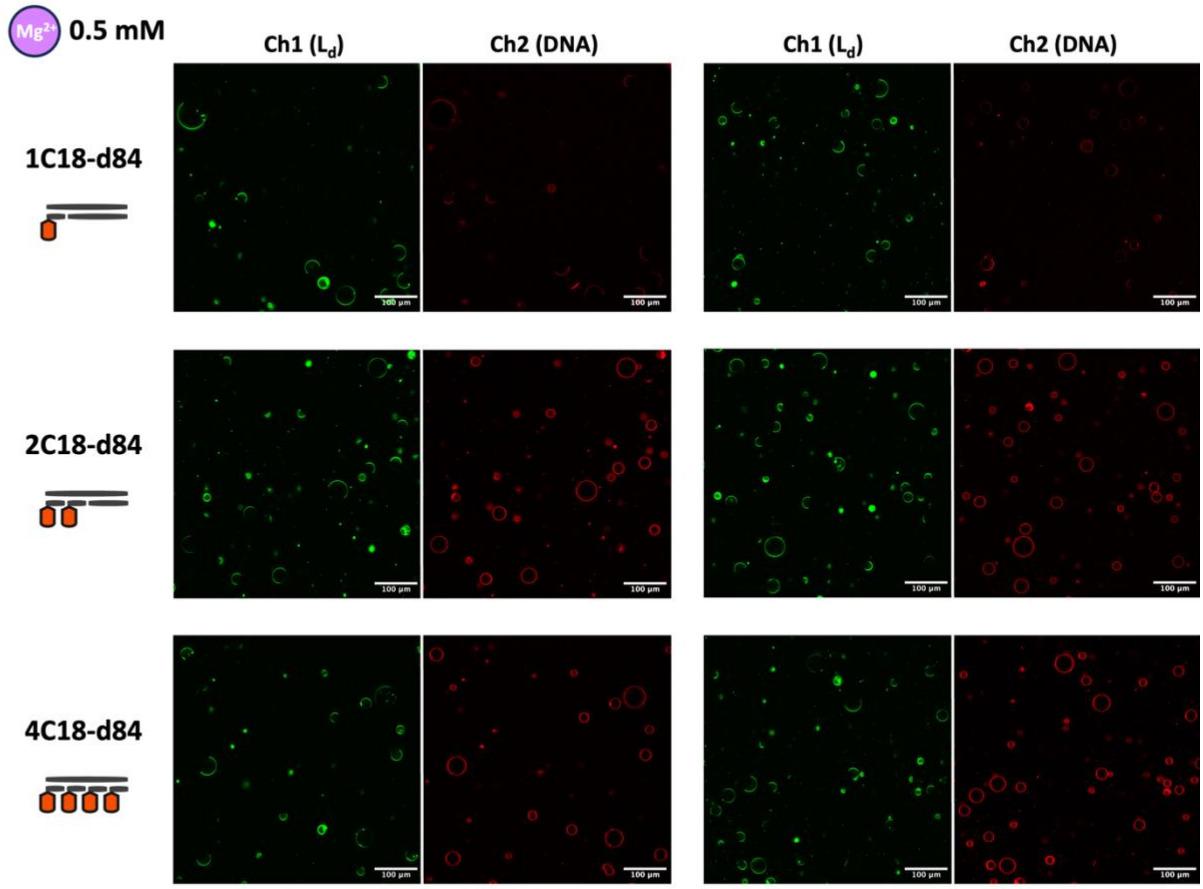

Figure S12. Confocal images of multivalently modified DNA (84 bp, 1–4-C18) binding to PS-GUVs, qualitatively demonstrating binding and partitioning distributions. Shown (top to bottom): 1-C18, 2-C18, and 4-C18 modifications. Brightness for all images was increased by 50% for visualization of phase selectivity; direct signal intensity comparisons with other modifications are invalid. Imaging parameters (laser intensity and gain) were standardized using the 4-α-tocopherol sample as a reference and optimized to achieve minimal saturation around GUVs. These randomly selected images supplement the representative micrographs in **Figure 2d (*C18 group*)**. Scale bar = 100 µm.

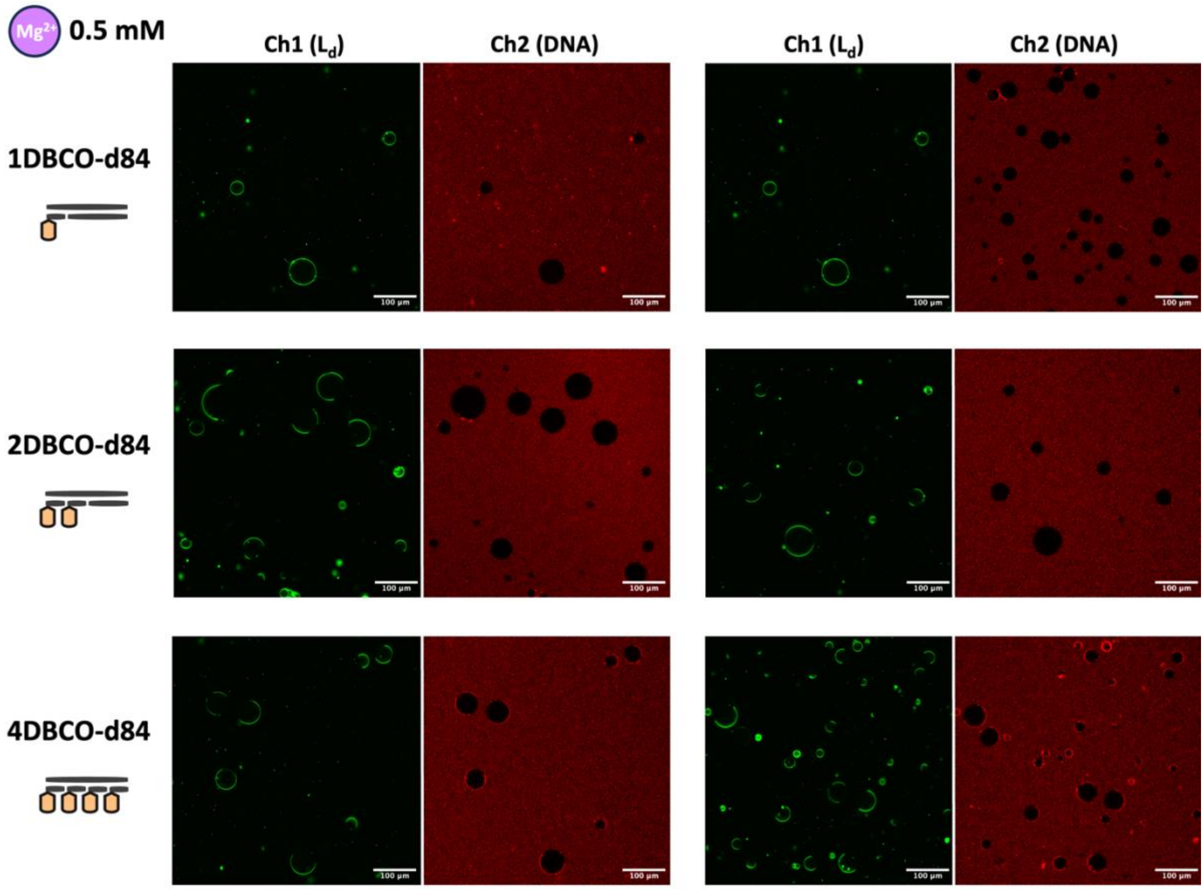

Figure S13. Confocal images of multivalently modified DNA (84 bp, 1–4-DBCO) binding to PS-GUVs, qualitatively demonstrating binding and partitioning distributions. Shown (top to bottom): 1-DBCO, 2-DBCO, and 4-DBCO modifications. Brightness for all images was increased by 90% for visualization of phase selectivity; direct signal intensity comparisons with other modifications are invalid. Imaging parameters (laser intensity and gain) were standardized using the 4-α-tocopherol sample as a reference and optimized to achieve minimal saturation around GUVs. These randomly selected images supplement the representative micrographs in **Figure 2d (*DBCO group*)**. Scale bar = 100 µm.

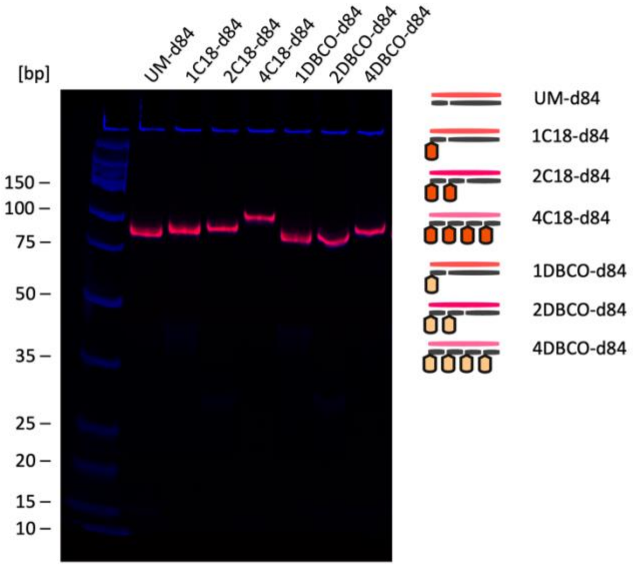

Figure S14. Polyacrylamide gel electrophoresis analysis of unmodified and anchor-modified 84 bp long DNA duplex labelled with Cy5 at 2 mM Mg^{2+} and schematic drawings illustrating the corresponding strands described in Table S1 and Figure S1 of the assembled structures.

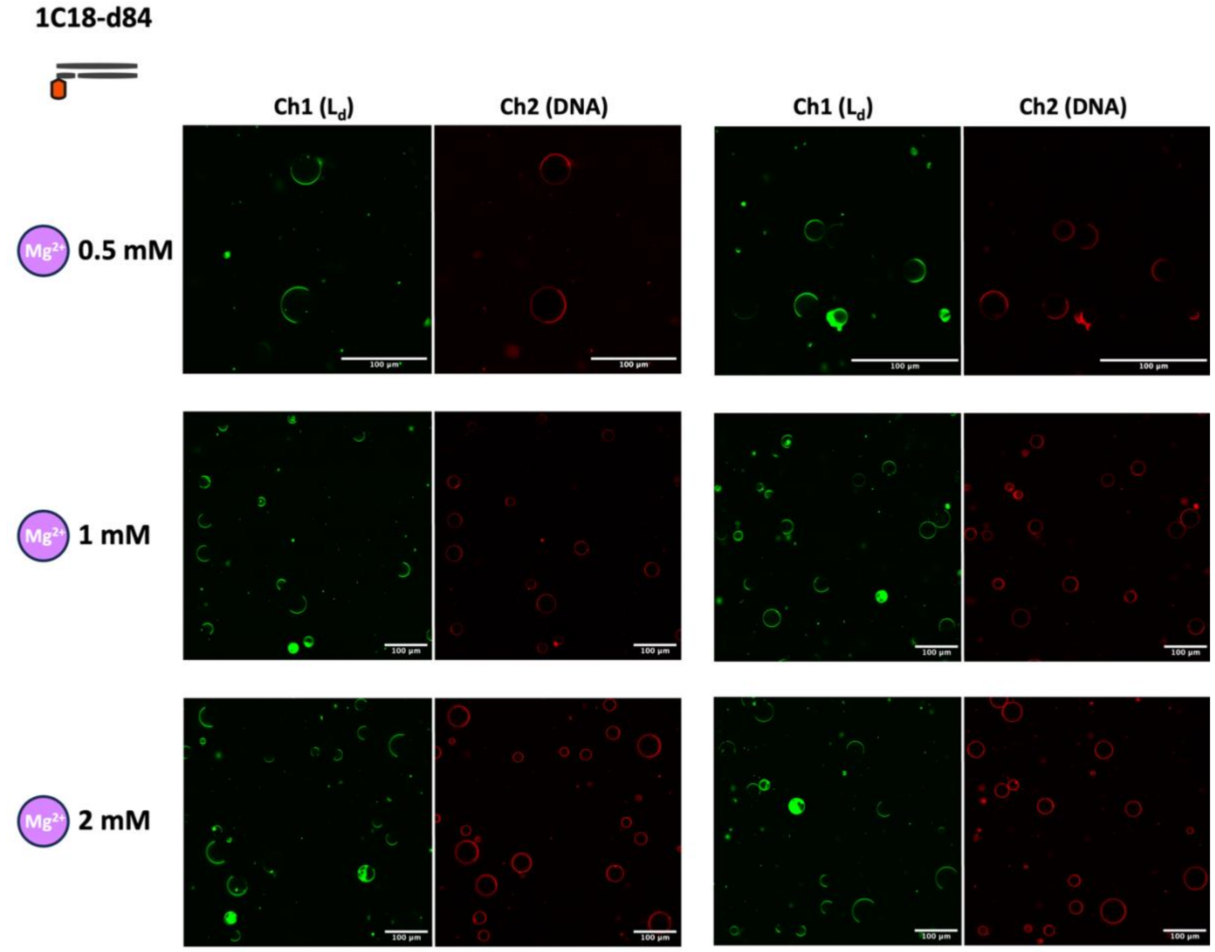

Figure S15. Confocal images of 1-C18-modified DNA (84 bp) binding to PS-GUVs at incremental Mg^{2+} concentrations (0.5, 1, and 2 mM), qualitatively illustrating binding and partitioning behavior. Brightness for the 0.5 mM Mg^{2+} images was increased by 50% to enhance phase selectivity visualization; direct comparisons of signal intensity across Mg^{2+} concentrations (see quantitative analysis in **Figure 3b**) are not valid. Imaging parameters (laser intensity and gain) were standardized using the 1-C18 reference sample in 2 mM Mg^{2+}, optimized to achieve minimal saturation near GUVs. These randomly selected images supplement the representative micrographs in **Figure 3a** (***1-C18 group***). Scale bar = 100 μm.

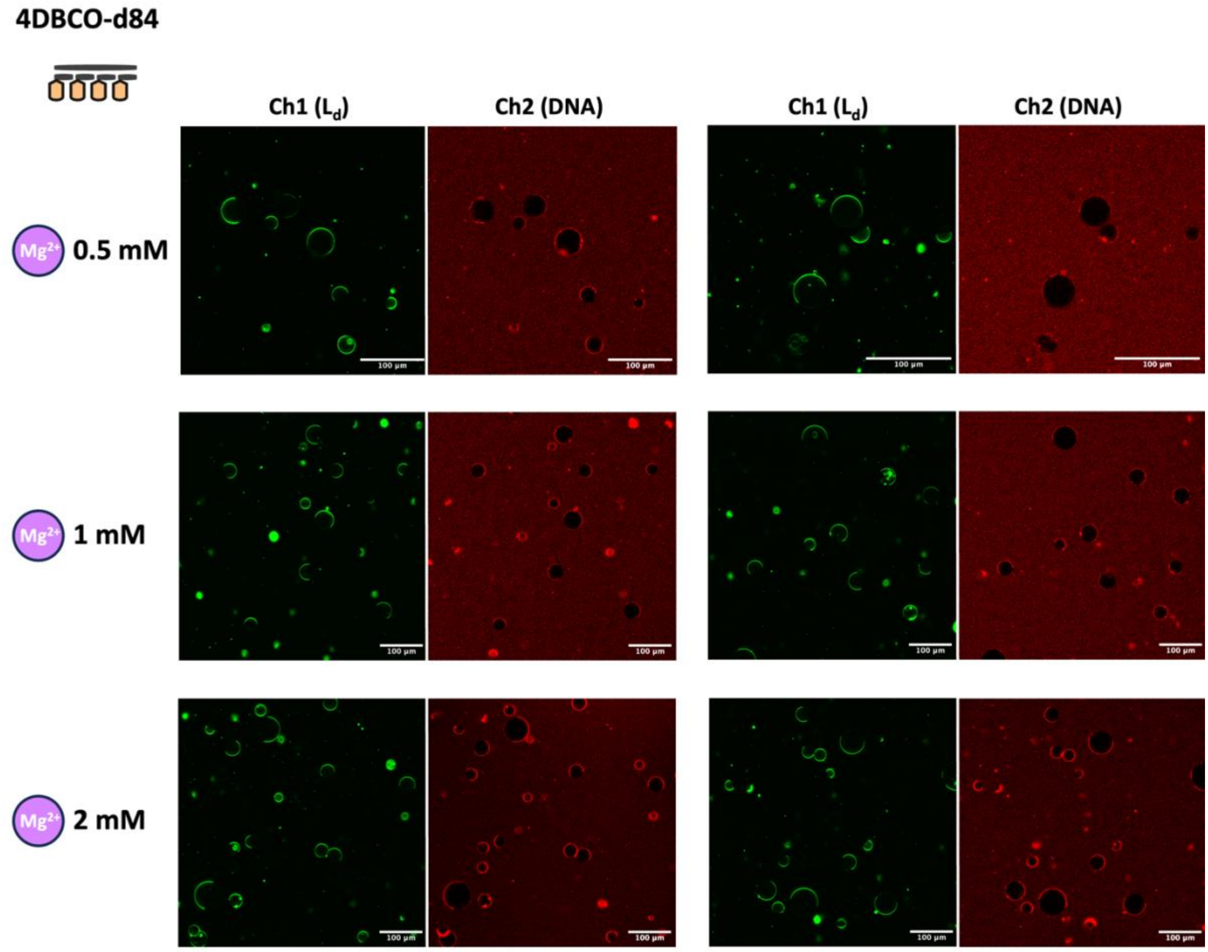

Figure S16. Confocal images of 4-DBCO-modified DNA (84 bp) binding to PS-GUVs at incremental Mg^{2+} concentrations (0.5, 1, and 2 mM), qualitatively demonstrating binding and partitioning behavior. Brightness for the 0.5, 1, and 2 mM Mg^{2+} images was increased by 90%, 80%, and 70%, respectively (evident from elevated background signals), to improve phase selectivity visualization; direct comparisons of signal intensity across Mg^{2+} concentrations (see quantitative analysis in **Figure 3b**) are not valid. Imaging parameters (laser intensity and gain) were standardized using the 1-C18 reference sample in 2 mM Mg^{2+}, optimized to achieve minimal saturation near GUVs. These randomly selected images supplement the representative micrographs in **Figure 3a (*4-DBCO group*)**. Scale bar = 100 µm.

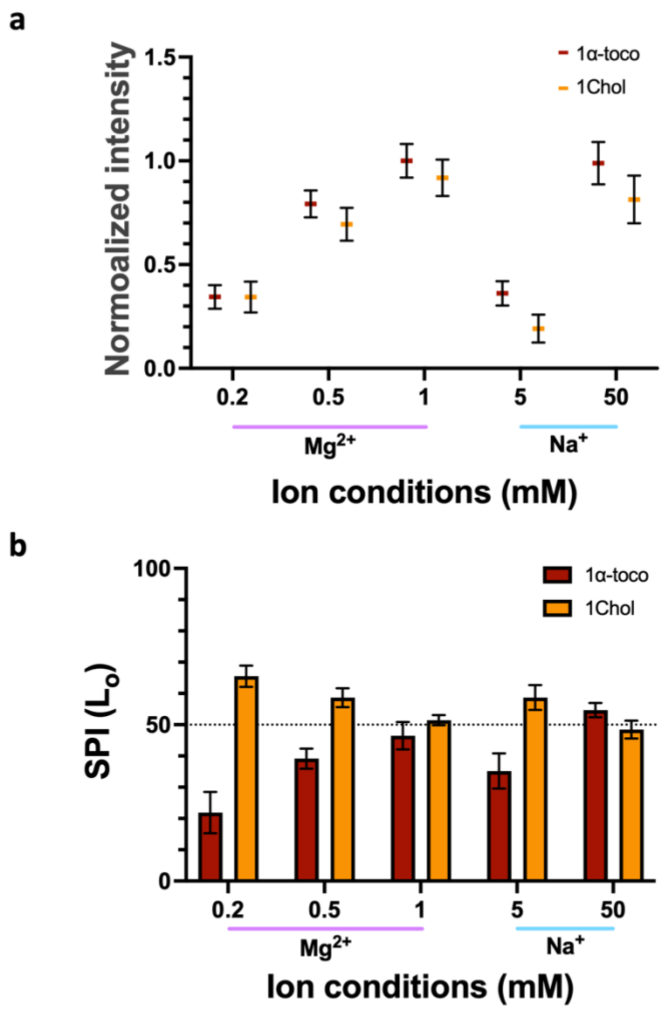

Figure S17. (a) Quantitative analysis of attachment efficiency for single α-tocopherol-modified (1-α-toco) and single cholesterol-modified (1-chol) DNA (84 bp) to PS-GUVs under varying ionic conditions (0.5–2 mM Mg^{2+}, 0.5 mM or 50 mM Na^{+}). Binding strength correlates positively with divalent Mg^{2+} concentration. Fluorescence signal intensities were normalized to 1-α-toco-modified DNA in 1 mM Mg^{2+} (reference value = 1). Data points represent mean values with 95% confidence intervals (CI). (b) Selective Partitioning Index (SPI) quantifying phase preference (L_o vs. L_d), revealing reduced Mg^{2+} concentration destabilizing strong hydrophobic anchors but enhancing phase selectivity due to the diminished electrostatic bridging. Error bars derived from two replicates (≥20 vesicles per replicate).

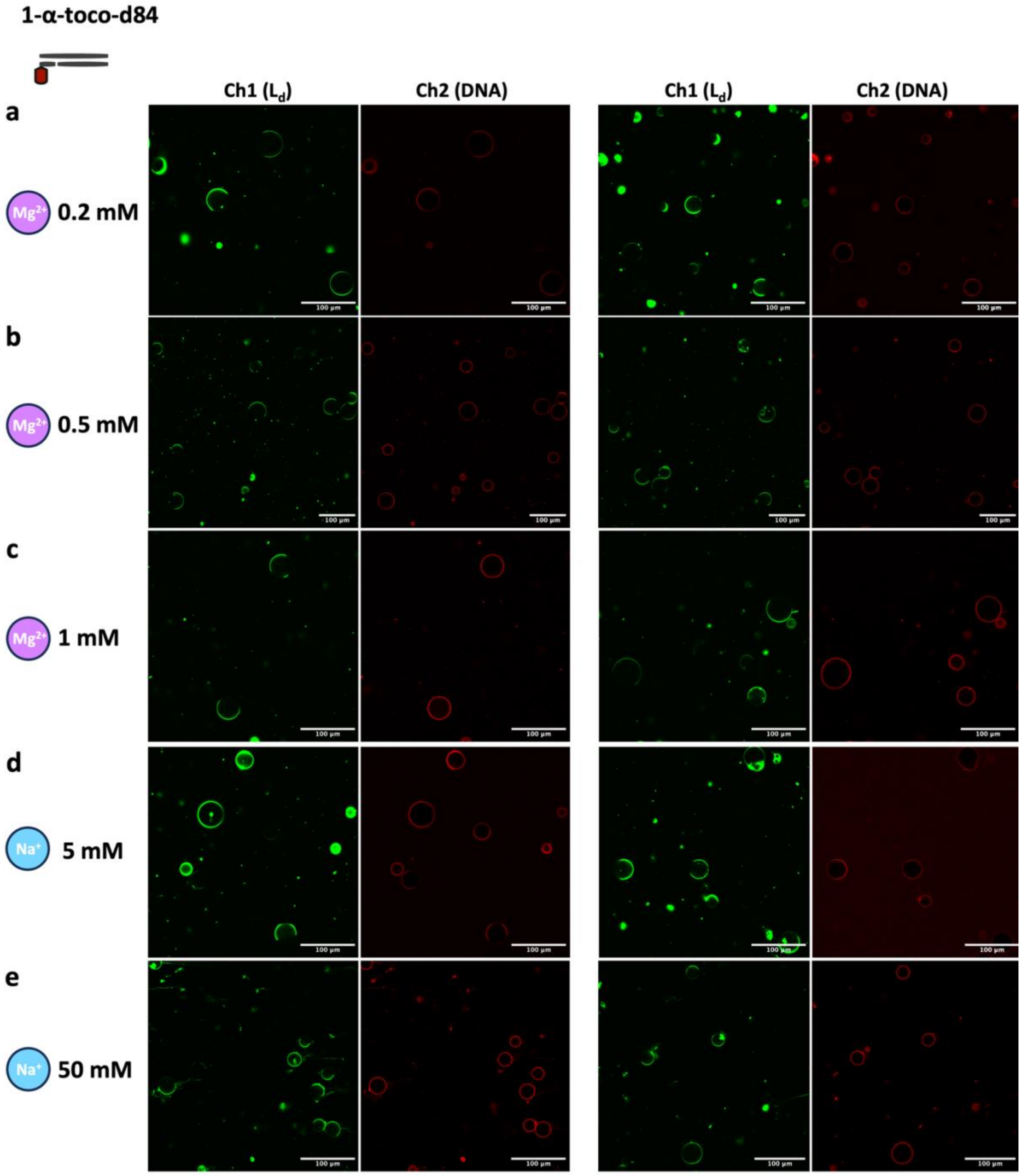

Figure S18. Confocal images of 1-α-toco-modified (84 bp) binding to PS-GUVs under varying ionic conditions: (a–c) 0.2–1 mM Mg^{2+} and (d, e) 5/50 mM Na^{+}, qualitatively illustrating ion-concentration-dependent binding and partitioning behavior. GUV in channel 1 confirms that stable GUV morphology and L_o/L_d phase separation at 0.2–2 mM Mg^{2+} and 5 mM Na^{+}, but tubule formation was observed at 50 mM Na^{+} (panel e), indicating partial membrane disruption. Brightness for the 0.2 mM Mg^{2+} and 5 mM Na^{+} images was increased by 30% to enhance phase selectivity visualization; direct comparisons of signal intensity (see quantitative analysis in **Figure S17a**) are not valid. Imaging parameters (laser intensity and gain) were standardized using the 1-α-toco reference sample in 1 mM Mg^{2+}, optimized to achieve minimal saturation near GUVs.These randomly selected images supplement the quantitative analysis in **Figure S17 (*strong anchor group: 1-α-toco*)**. Scale bar = 100 μm.

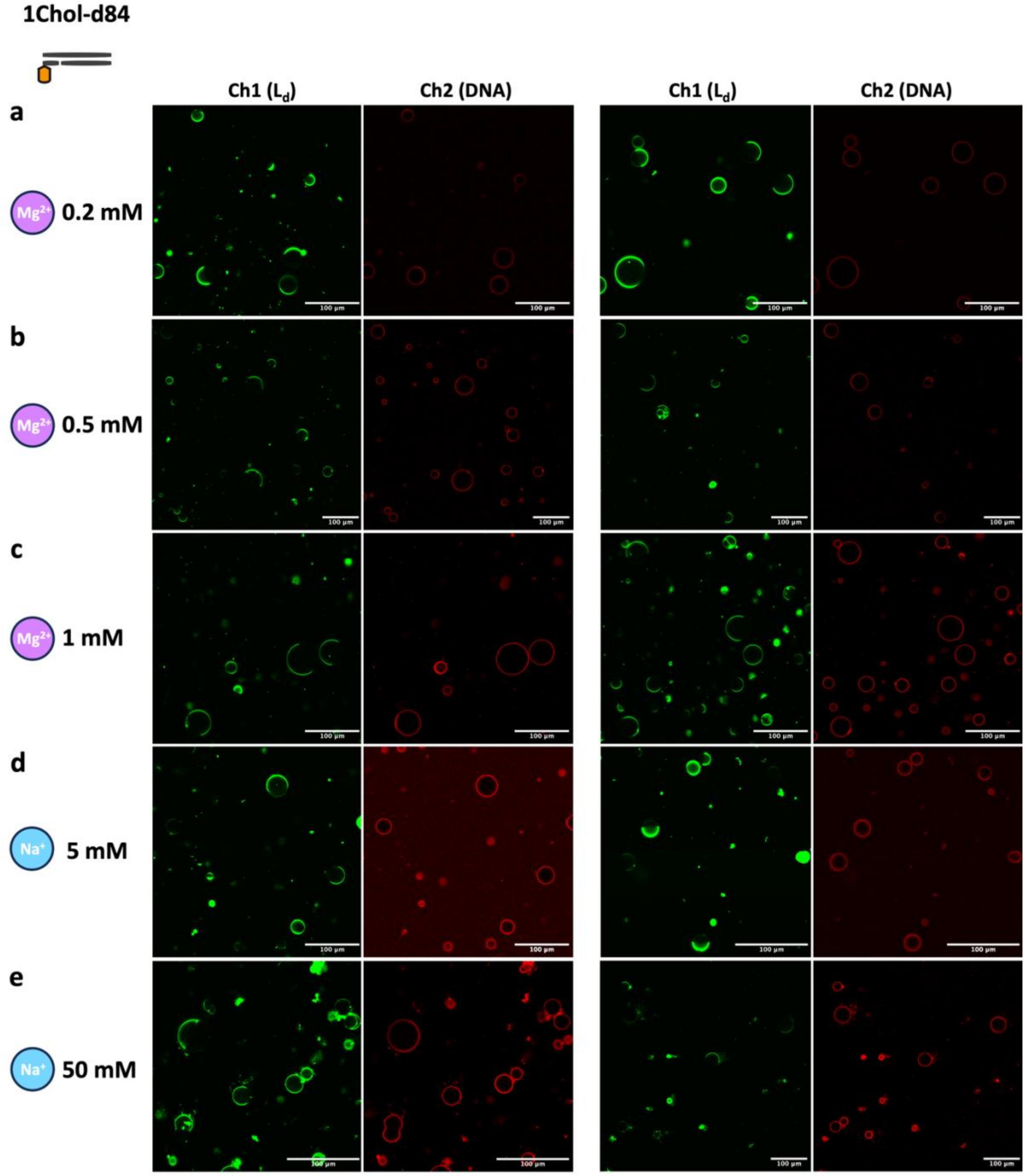

Figure S19. Confocal images of 1-Chol-modified DNA (84 bp) binding to PS-GUVs under varying ionic conditions: (a–c) 0.2–1 mM Mg^{2+} and (d–e) 5/50 mM Na^+, qualitatively demonstrating ion-concentration-dependent binding and partitioning behavior. GUVs in channel 1 verify stable morphology and L_o/L_d domains at 0.2–2 mM Mg^{2+} and 5 mM Na^+, with tubule formation observed at 50 mM Na^+ (panel e), suggesting partial membrane disruption. Brightness for the 0.2 mM Mg^{2+} and 5 mM Na^+ images was increased by 30% and 50%, respectively, to enhance phase selectivity visualization; direct comparisons of signal intensity (see quantitative analysis in **Figure S17a**) are not valid. Imaging parameters (laser intensity and gain) were standardized using the 1-α-toco reference sample in 1 mM Mg^{2+}, optimized to achieve minimal saturation near GUVs. These randomly selected images supplement the quantitative analysis in **Figure S17 (*strong anchor group: 1-chol*)**. Scale bar = 100 µm.

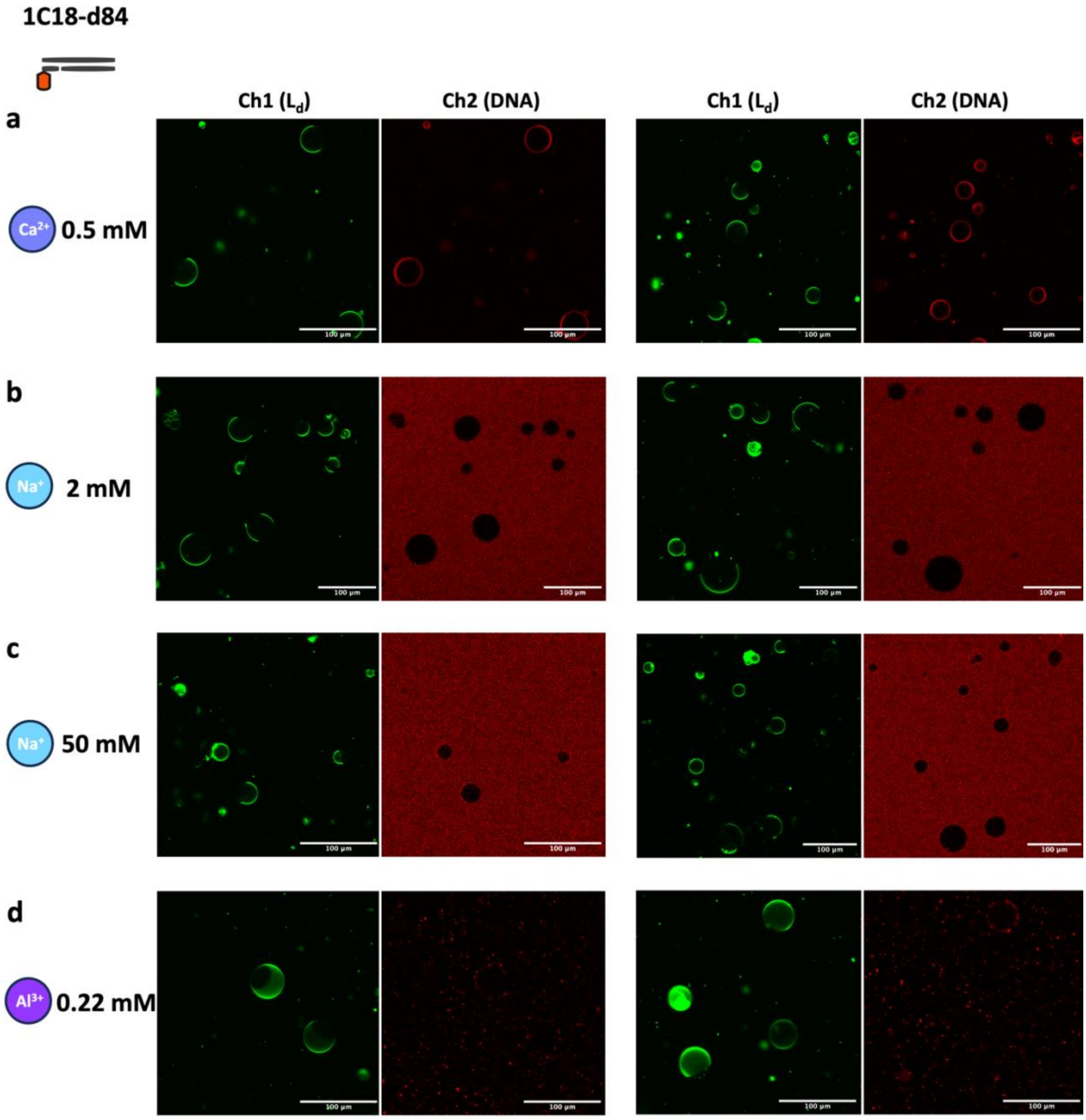

Figure S20. Confocal images of 1-C18-modified DNA (84 bp) binding to PS-GUVs under varying ionic conditions: (a) 0.5 mM Ca^{2+}, (b) 50 mM Na^{+}, (c) 50 mM or (d) Na^{+}, 0.22 mM Al^{3+}, ion-valency-dependent binding and partitioning. Ca^{2+} served as a stronger electrostatic bridging agent compared to Mg^{2+}. Monovalent Na^{+} failed to sustain binding unless at extreme electrostatic screening strength (50 mM), which induced weak, non-specific binding. Al^{3+} caused aggregation due to nucleic acid condensation without direct membrane damage, excluding from partitioning analysis. Brightness for the 0.5 mM Ca^{2+} and 2/50 mM Na^{+} images was increased by 50% and 90%, respectively, to enhance phase selectivity visualization; direct comparisons of signal intensity across other ionic conditions (see quantitative analysis in **Figure 3b**) are not valid. Imaging parameters (laser intensity and gain) were standardized using the 1-C18 reference sample in 2 mM Mg^{2+}, optimized to achieve minimal saturation near GUVs. These randomly selected images supplement the representative micrographs in **Figure 3c (*1-C18 group*)**. Scale bar = 100 µm.

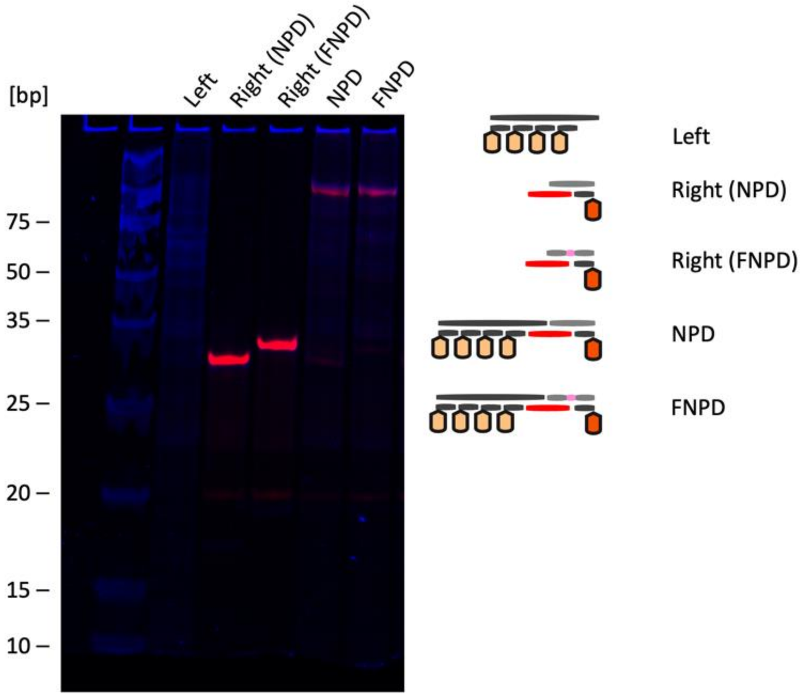

Figure S21. Polyacrylamide gel electrophoresis analysis of dual-anchor DNA nanostructures: NanoPhase DNA (NPD) (rigid spacing) and Flexible NanoPhase DNA (FNPD) (ssDNA linker). Strands are shown in Left (S6+4S1DBCO), Right (NPD) (S5+S7+S1C18), Right (FNPD) (S5F+S7+S1C18), NPD (Left+Right (NPD)), and FNPD (Left+Right (FNPD)), respectively.

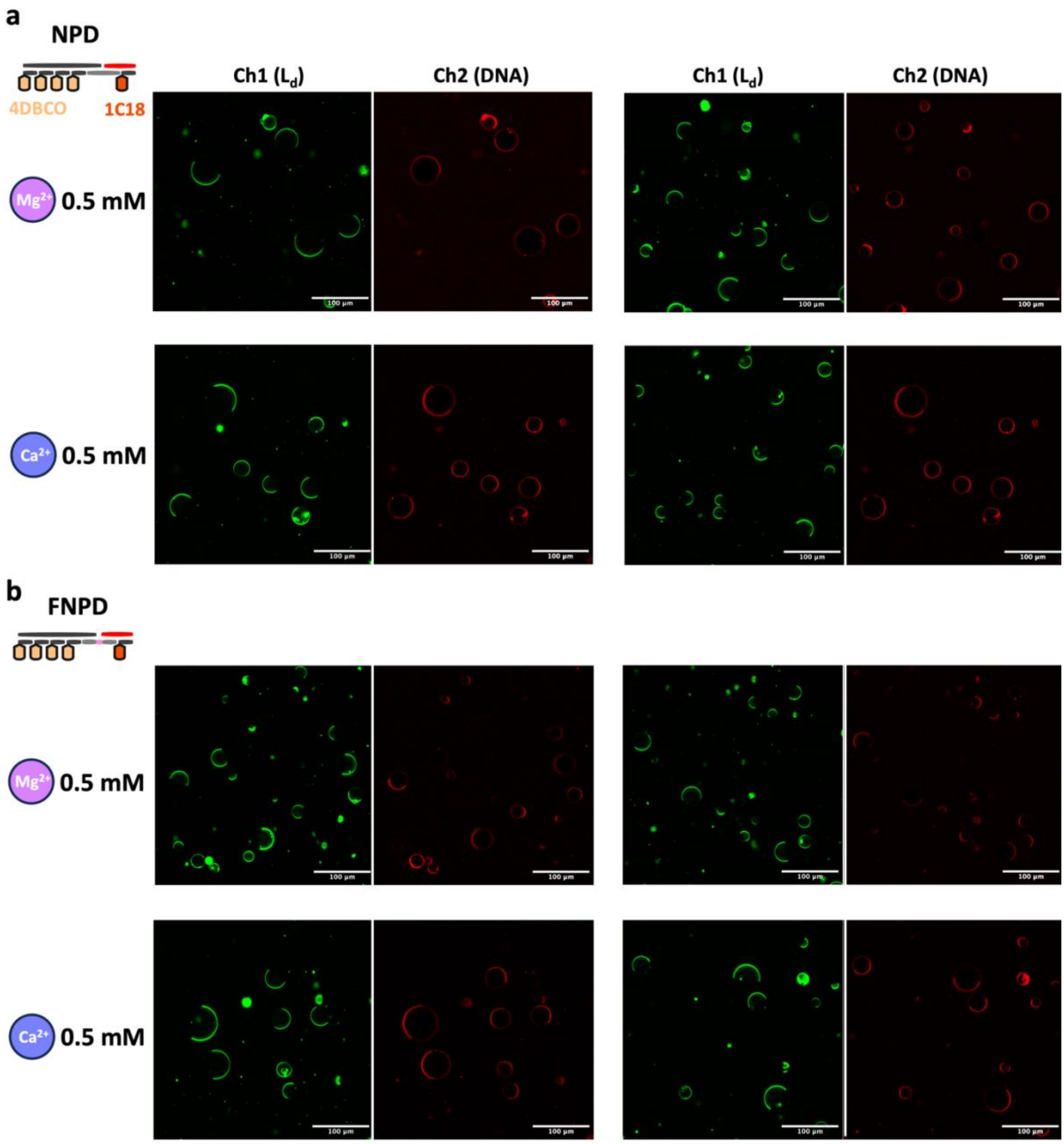

Figure S22. (a) Confocal microscopy images of NanoPhase DNA (NPD) and (b) Flexible NanoPhase DNA (FNPD) binding to PS-GUVs in 0.5 mM Mg^{2+} or Ca^{2+}, demonstrating preferential partitioning into liquid-ordered (L_o) domains (SPI ≈ 70–80%; **Figure 4c**). Despite DBCO's intrinsic affinity for liquid-disordered (L_d) regions, the strong Lo-phase selectivity of the 1-C18 anchor likely reflects its superior binding affinity (**Figure S9**). Imaging parameters (laser intensity and gain) were standardized using the NPD reference sample in 0.5 mM Ca^{2+}, optimized to achieve minimal saturation near GUVs. These randomly selected images supplement the representative micrographs in **Figure 4b**. Scale bar = 100 µm.

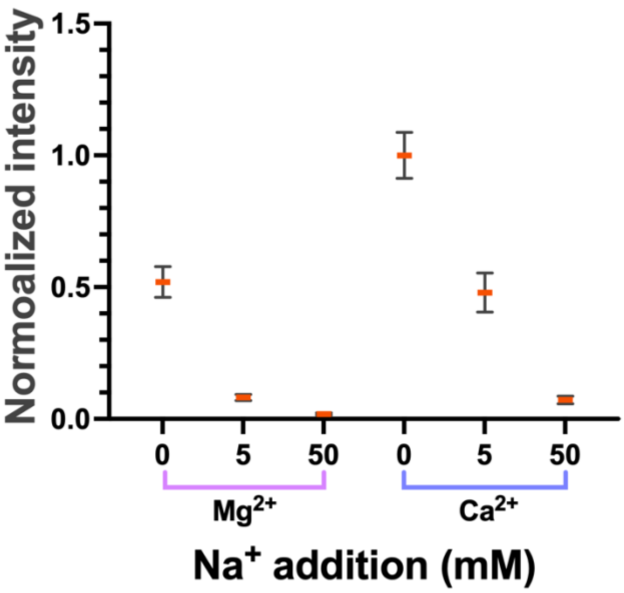

Figure S23. Attachment efficiency for 1-C18-modified DNA (84 bp) to PS-GUVs in pre-equilibrated 0.5 mM Mg^{2+} or Ca^{2+} with incremental Na^{+} (0–50 mM). Increasing Na^{+} weakened DNA attachment by competing with divalent ions (Mg^{2+}/Ca^{2+}), compromising electrostatic bridging. Ca^{2+} exhibited stronger charge screening efficiency than Mg^{2+}. Fluorescence signal intensities were normalized to the constructs' binding in 0.5 mM Ca^{2+} without Na^{+} addition (reference value = 1). Data points represent mean values with 95% confidence intervals (CI), derived from two replicates (≥20 vesicles per replicate).

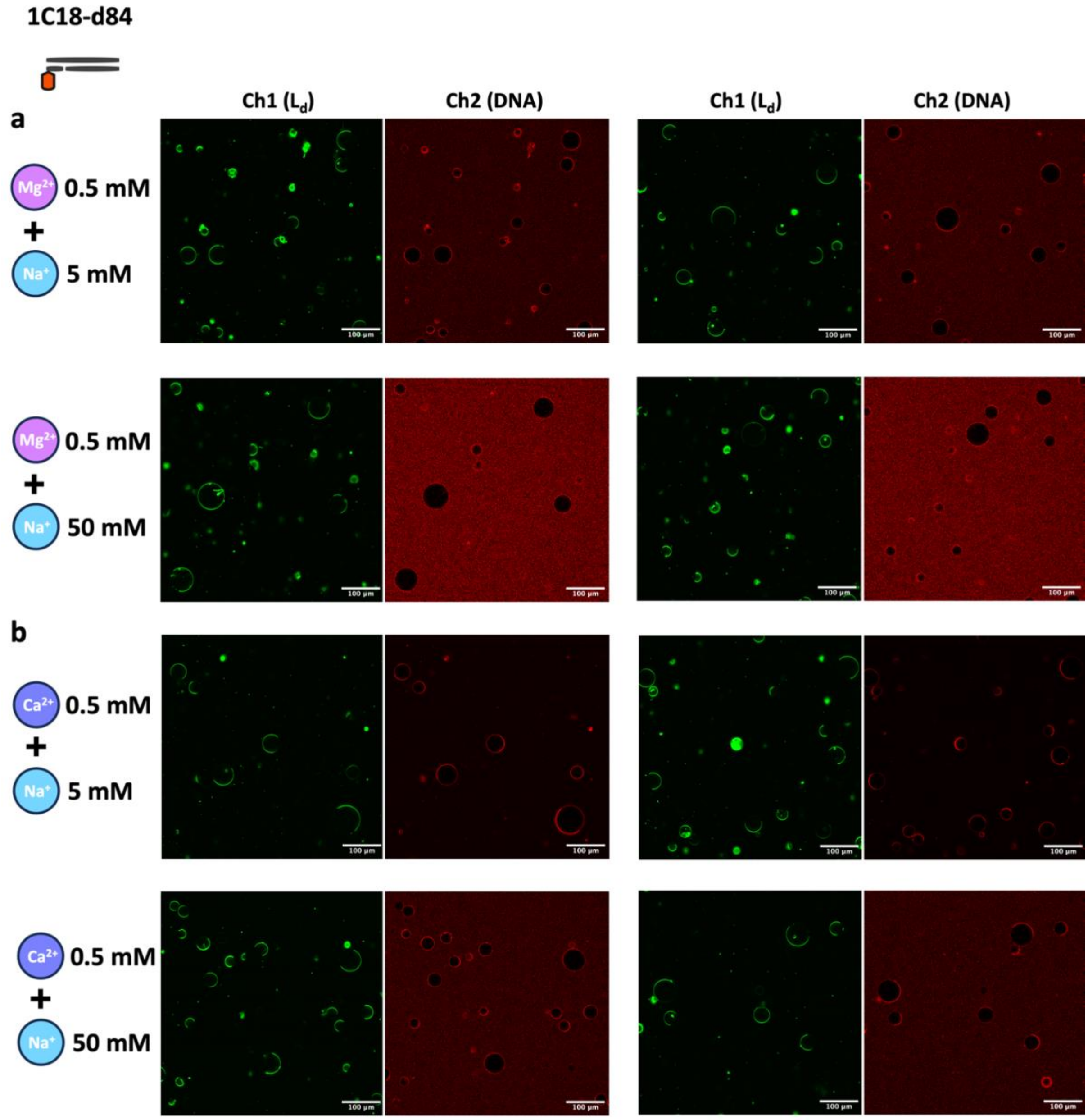

Figure S24. (a) Confocal images of 1-C18-modified DNA (84 bp) binding to PS-GUVs in pre-equilibrated 0.5 mM Mg^{2+} and (b) Ca^{2+} with incremental Na^{+} (0–50 mM), illustrating ion-dependent binding and partitioning. Na^{+} competition destabilized Mg^{2+}-mediated binding more readily than Ca^{2+}-mediated interactions, reflecting Ca^{2+}'s superior bridging efficiency and resilience, likely due to its lower charge density and higher electropositivity. Brightness adjustments: Ca^{2+} + 5/50 mM Na^{+} (+50%/80%) and Mg^{2+} + 5/50 mM Na^{+} (+80%/90%) enhance phase selectivity; direct comparisons of signal intensity are invalid (see quantitative analysis in **Figure S23**). Imaging parameters (laser intensity and gain) were standardized using the 1-C18 reference sample in 0.5 mM Ca^{2+}, optimized to achieve minimal saturation near GUVs. These randomly selected images supplement the representative micrographs in **Figure 4d**. Scale bar = 100 μm.

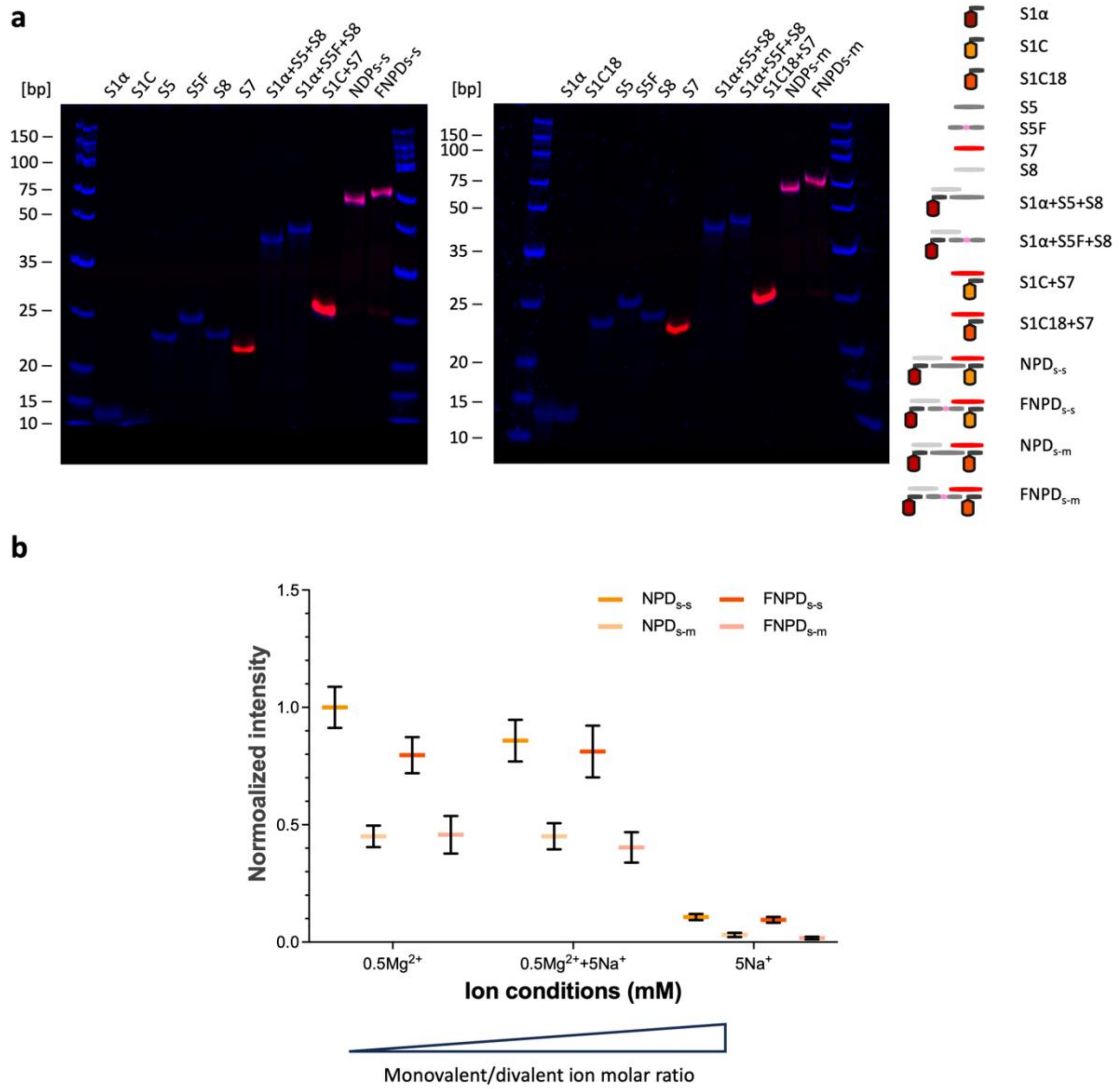

Figure S25. Analysis of dual-anchor DNA nanostructures. (a) PAGE analysis: NanoPhase DNA (NPD) (rigid spacing) and Flexible NanoPhase DNA (FNPD) (ssDNA linker), pairing α-tocopherol (strong L_d-preferring) with either cholesterol or C18 (strong or moderate L_o-preferring), termed (F)$NPD_{s\text{-}s}$ or (F)$NPD_{s\text{-}m}$, respectively. (b) Quantitative analysis of attachment efficiency for all rigid or flexible Nanophrase DNA (NPD/FNPD) (84 bp) to PS-GUVs under varying ionic conditions (0.5 mM Mg^{2+}, 0.5 mM Mg^{2+} + 5 mM Na^{+}, and 5 mM Na^{+}). Binding strength correlates negatively with the monovalent-to-divalent ion ratio. Fluorescence signal intensities were normalized to NPDs-s in 0.5 mM Mg^{2+} (reference value = 1). Data points represent mean values with 95% confidence intervals (CI).

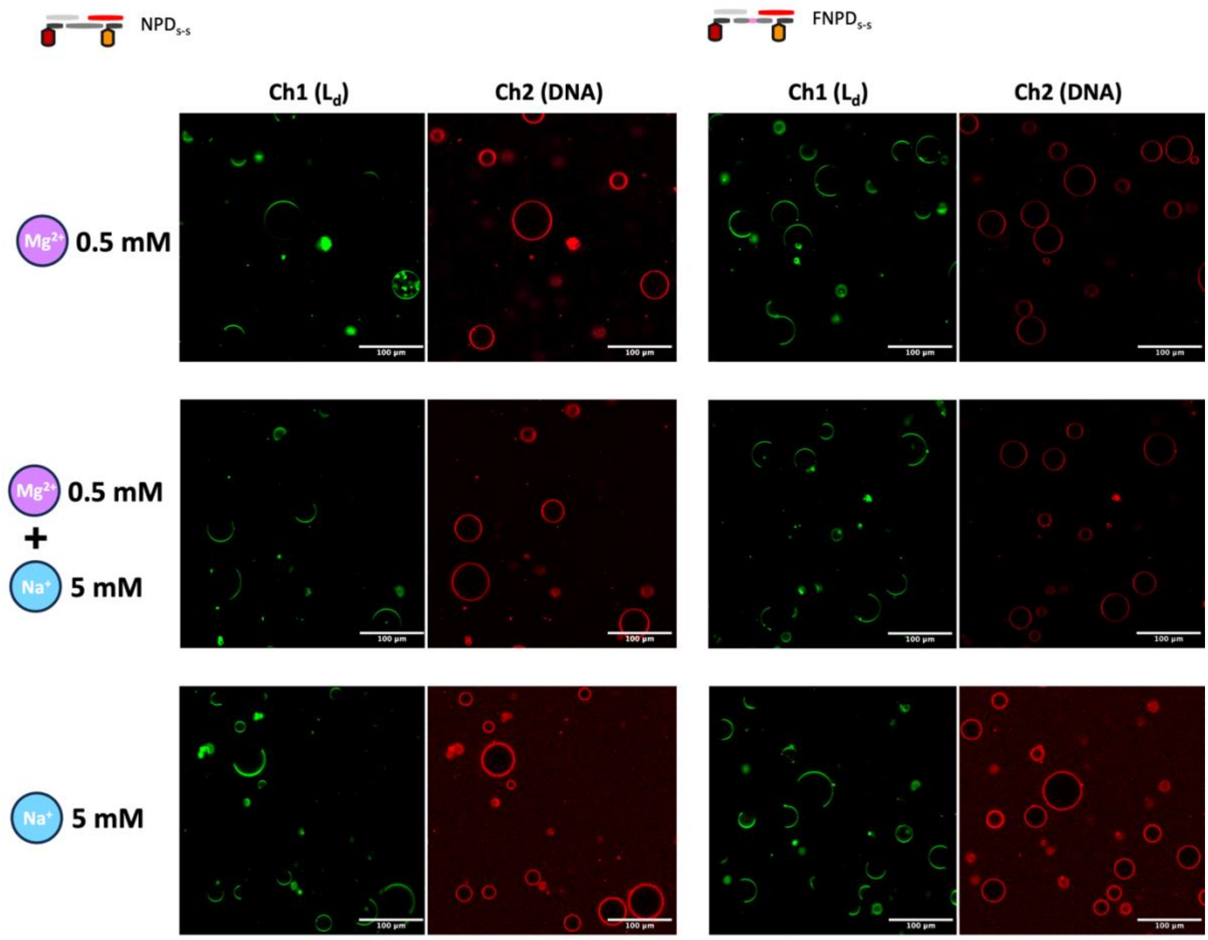

Figure S26. Confocal images of NPD$_{s-s}$ and FNPD$_{s-s}$ binding to PS-GUVs under varying ionic conditions (0.5 mM Mg^{2+}, 0.5 mM Mg^{2+} + 5 mM Na^+, and 5 mM Na^+), demonstrating strong dependency on the *monovalent-to-divalent ion ratio*. Competitive Na^+ ions neutralized phase preference by disrupting divalent bridging and amplifying anchor competition, implying divalent bridging is more effective in tightly packed liquid-ordered (L_o) domains than in disordered (L_d) phases. This observation suggests that divalent bridging is more effective in the tightly packed lipids of the L_o phase compared to the loosely organized L_d phase. Brightness for the 5 mM Na^+ images was increased by 80% for visualization; direct signal comparisons are invalid (see quantitative analysis in **Figure 5b** and **Figure S25**). Imaging parameters were standardized using the NPDs-s reference sample in 0.5 mM Mg^{2+}, optimized to achieve minimal saturation near GUVs. These images supplement **Figure 5b**. Scale bar = 100 µm.

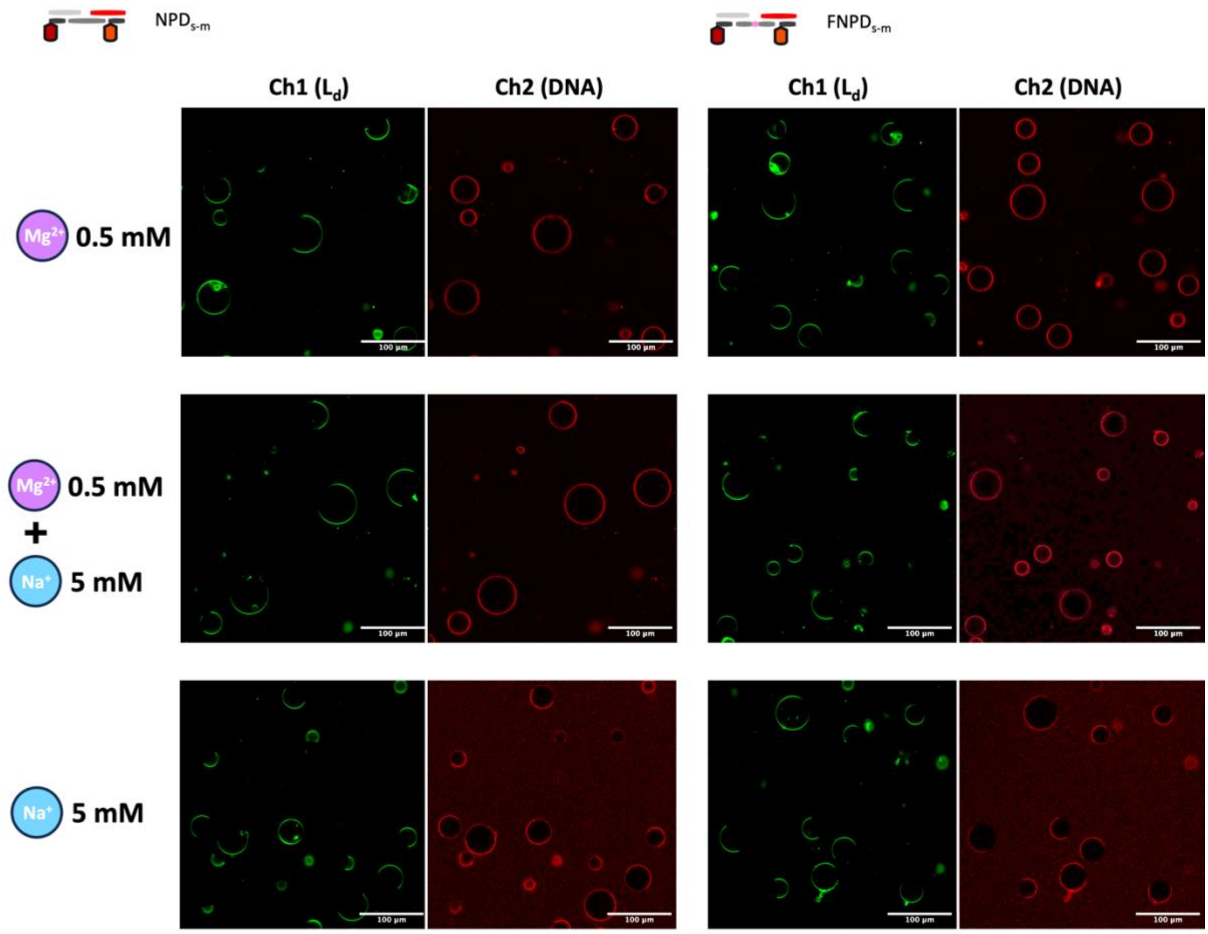

Figure S27. Confocal images of $NPD_{s\text{-}m}$ and $FNPD_{s\text{-}m}$ binding to PS-GUVs under varying ionic conditions (0.5 mM Mg^{2+}, 0.5 mM Mg^{2+} + 5 mM Na^{+}, and 5 mM Na^{+}), revealing strong dependency on the *monovalent-to-divalent ion ratio*. Competitive Na^{+} diminished the C18 anchor's contribution to liquid-ordered (L_o) localization, enhancing L_d-phase preference driven exclusively by α-tocopherol. Brightness for the 5 mM Na^{+} images was increased by 80%,, to enhance visualization; direct signal comparisons are invalid (see quantitative analysis in **Figure 5b** and **Figure S25**). Imaging parameters (laser intensity and gain) were standardized using the NPDs-s reference sample in 0.5 mM Mg^{2+}, optimized to achieve minimal saturation near GUVs. These randomly selected images supplement the quantitative analysis in **Figure 5b**. Scale bar = 100 µm.

Table S1: Sequences and structures of oligonucleotides to assemble DNA duplexes presented in **Figure S1**. L. and Mod. stand for length and modification, respectively.

Structure	Strand	Sequence (5' → 3')	L.	Mod.
	S1	GATGACGGTATAGCAAGTGTG	21	-
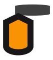	S1C	GATGACGGTATAGCAAGTGTG	21	Chol-TEG
	S2	CACACTTGCTATACCGTCATC	21	Cy5
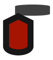	S1α	GATGACGGTATAGCAAGTGTG	21	α-toco-TEG
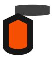	S1C18	GATGACGGTATAGCAAGTGTG	21	C18
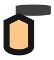	S1DBCO	GATGACGGTATAGCAAGTGTG	21	DBCO-TEG
	S3a	AGTGAACAATTATATGGGCATGTTGGGAGGAGTGTGG AGCCA TTAACAGCTTGATGATTGACC	63	-
	S4a	GGTCAATCATCAAGCTGTTAATGGCTCCACACTCCTCCC AACATGCCCATATAATTGTTCACTCACACTTGCTATACC GTCATC	84	Cy5
	S3b	GTTGGGAGGAGTGTGGAGCCATTAACAGCTTGATGATT GACC	42	-
	S4b	GGTCAATCATCAAGCTGTTAATGGCTCCACACTCCTCCC AACCACACTTGCTATACCGTCATCCACACTTGCTATACC GTCATC	84	Cy5
	S4c	CACACTTGCTATACCGTCATCCACACTTGCTATACCGTC ATCCACACTTGCTATACCGTCATCCACACTTGCTATACC GTCATC	84	Cy5
	S5	TTAACAGCTTGATGATTGACCCTTCCCTGGTGAGAAGTC AGC	42	-
	S5F	TTAACAGCTTGATGATTGACCTTTTTCTTCCCTGGTGAG AAGTCAGC	47	-
	S6	GGTCAATCATCAAGCTGTTAACACACTTGCTATACCGTC ATCCACACTTGCTATACCGTCATCCACACTTGCTATACC GTCATCCACACTTGCTATACCGTCATC	105	-
	S7	CACACTTGCTATACCGTCATCGCTGACTTCTCACCAGGG AAG	42	Cy5
	S8	GGTCAATCATCAAGCTGTTAACACACTTGCTATACCGTC ATC	42	-

Table S2. Information on lipids used (details obtained from Avanti® Polar Lipids), including DMPC LUVs and DOPC/DPPC/cholesterol GUVs mixture compositions.

Lipids	Chemical structure	Phase transition temperature	Ratio (%)
14:0 PC (DMPC)	1,2-dimyristoyl-sn-glycero-3-phosphocholine 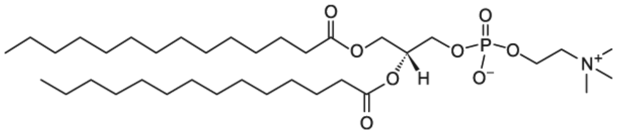	24.5°C	N/A
18:1c9 PC (DOPC)	1,2-dioleoyl-sn-glycero-3-phosphocholine 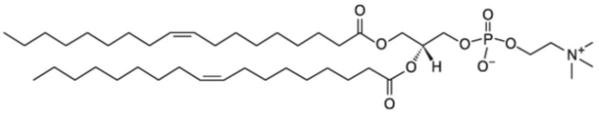	-17°C	39.75
16:0 PC (DPPC)	1,2-dipalmitoyl-sn-glycero-3-phosphocholine 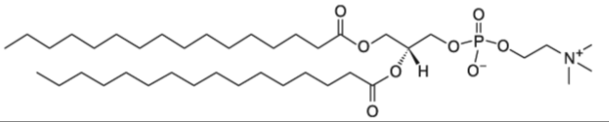	41°C	39.75
18:1 Liss Rhod PE	1,2-dioleoyl-sn-glycero-3-phosphoethanolamine-N-(lissamine rhodamine B sulfonyl) (ammonium salt) 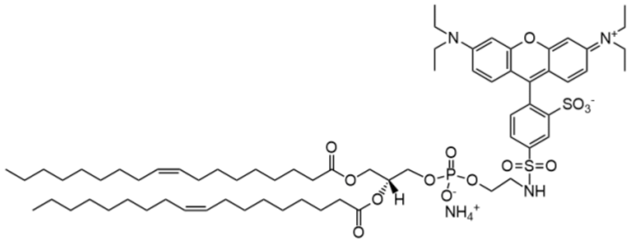	-16°C	0.5
Cholesterol		N/A	20

References

(1) Rubio-Sánchez, R.; Barker, S. E.; Walczak, M.; Cicuta, P.; Michele, L. D. A Modular, Dynamic, DNA-Based Platform for Regulating Cargo Distribution and Transport between Lipid Domains. *Nano Lett.* **2021**, *21* (7), 2800–2808.

(2) Rong, W.; Li, Z.; Zhang, W.; Sun, L. An Improved Canny Edge Detection Algorithm. In *2014 IEEE International Conference on Mechatronics and Automation*; IEEE: Tianjin, China, 2014; pp 577–582. https://doi.org/10.1109/ICMA.2014.6885761.

(3) Yuen, H.; Princen, J.; Illingworth, J.; Kittler, J. Comparative Study of Hough Transform Methods for Circle Finding. *Image Vis. Comput.* **1990**, *8* (1), 71–77. https://doi.org/10.1016/0262-8856(90)90059-E.

(4) Ye, H.; Shang, G.; Wang, L.; Zheng, M. A New Method Based on Hough Transform for Quick Line and Circle Detection. In *2015 8th International Conference on Biomedical Engineering and Informatics (BMEI)*; IEEE: Shenyang, China, 2015; pp 52–56. https://doi.org/10.1109/BMEI.2015.7401472.

(5) Pawley, J. *Handbook of Biological Confocal Microscopy*; Springer Science & Business Media, 2006; Vol. 236.

(6) *Chemicalize - Instant Cheminformatics Solutions*. Chemicalize. https://chemicalize.com (accessed 2024-03-14).
